# Association between *in vivo* bone formation and *ex vivo* migratory capacity of human bone marrow stromal cells

**DOI:** 10.1186/s13287-015-0188-9

**Published:** 2015-10-08

**Authors:** Rikke K. Andersen, Walid Zaher, Kenneth H. Larsen, Nicholas Ditzel, Katharina Drews, Wasco Wruck, James Adjaye, Basem M. Abdallah, Moustapha Kassem

**Affiliations:** Department of Endocrinology and Metabolism, University Hospital of Odense, Odense, Denmark; Stem Cell Unit, Department of Anatomy, College of Medicine, King Saud University, Riyadh, Saudi Arabia; Department of Vertebrate Genomics, Molecular Embryology and Aging Group, Max Planck Institute for Molecular Genetics, Berlin, Germany; Research and Regenerative Medicine, Faculty of Medicine, Heinrich Heine University, Duesseldorf, Germany; Faculty of Science, Helwan University, Cairo, Egypt; Danish Stem Cell Center (DanStem), Faculty of Health Sciences, University of Copenhagen, Copenhagen, Denmark

## Abstract

**Introduction:**

There is a clinical need for developing systemic transplantation protocols for use of human skeletal stem cells (also known bone marrow stromal stem cells) (hBMSC) in tissue regeneration. In systemic transplantation studies, only a limited number of hBMSC home to injured tissues suggesting that only a subpopulation of hBMSC possesses “homing” capacity. Thus, we tested the hypothesis that a subpopulation of hBMSC defined by ability to form heterotopic bone *in vivo*, is capable of homing to injured bone.

**Methods:**

We tested *ex vivo* and *in vivo* homing capacity of a number of clonal cell populations derived from telomerized hBMSC (hBMSC-TERT) with variable ability to form heterotopic bone when implanted subcutaneously in immune deficient mice. *In vitro* transwell migration assay was used and the *in vivo* homing ability of transplanted hBMSC to bone fractures in mice was visualized by bioluminescence imaging (BLI). In order to identify the molecular phenotype associated with enhanced migration, we carried out comparative DNA microarray analysis of gene expression of hBMSC-derived high bone forming (HBF) clones versus low bone forming (LBF) clones.

**Results:**

HBF clones were exhibited higher *ex vivo* transwell migration and following intravenous injection, better *in vivo* homing ability to bone fracture when compared to LBF clones. Comparative microarray analysis of HBF versus LBF clones identified enrichment of gene categories of chemo-attraction, adhesion and migration associated genes. Among these, platelet-derived growth factor receptor (PDGFR) α and β were highly expressed in HBF clones. Follow up studies showed that the chemoattractant effects of PDGF *in vitro* was more enhanced in HBF compared to LBF clones and this effect was reduced in presence of a PDGFRβ-specific inhibitor: SU-16 f. Also, PDGF exerted greater chemoattractant effect on PDGFRβ^+^ cells sorted from LBF clones compared to PDGFRβ^-^ cells.

**Conclusion:**

Our data demonstrate phenotypic and molecular association between *in vivo* bone forming ability and migratory capacity of hBMSC. PDGFRβ can be used as a potential marker for the prospective selection of hBMSC populations with high migration and bone formation capacities suitable for clinical trials for enhancing bone regeneration.

**Electronic supplementary material:**

The online version of this article (doi:10.1186/s13287-015-0188-9) contains supplementary material, which is available to authorized users.

## Introduction

Human skeletal stem cells (also known as human bone marrow-derived stromal cells (hBMSC)) are adult multipotent stem cells located in the bone marrow perivascular niche and are recruited to bone formation sites during bone remodeling [1]. During recent years, hBMSC have been tested in a number of clinical trials for their ability to enhance tissue repair including tissue regeneration where hBMSC were injected locally at the sites of tissue injury; for example, bone fracture [2–4] or ischemic myocardium [5–8]. However, systemic intravenous infusion is more suitable for clinical cell transplantation and is employed for hematopoietic stem cell (HSC) transplantation with success and where HSCs, following homing from systemic circulation to bone marrow, engraft and initiate hematopoiesis [9].

Several studies have demonstrated that systemically injected bone marrow-derived stromal cells (BMSC) can home to damaged tissues in animal models of brain injury [10], skeletal disorders [11–13], and acute radiation syndrome [14, 15]. However, the number of BMSC that home and engraft in injured tissues is usually small and most of the infused BMSC get entrapped in the lungs [16, 17]. The explanation for these phenomena is still missing because the mechanisms governing migration of BMSC to injured tissues are poorly understood [18].

Cultured hBMSC are a heterogeneous population of cells that when analyzed at a clonal level exhibit variations in cell morphology, proliferation, and differentiation capacity [19, 20]. Recently, we have also demonstrated that clonal heterogeneity of the hBMSC population reflects functional heterogeneity with respect to cell capacity for osteoblast adipocyte differentiation or immune functions [21, 22].

Here we hypothesized the existence of clonal heterogeneity in the ability of hBMSC to home to injured tissues (e.g., bone fractures) and that hBMSC with good bone-forming capacity will be more efficient at homing to bone fracture sites. To test this hypothesis, we examined the *ex vivo* and *in vivo* migratory capacity of a number of clonal cell populations isolated from telomerized hBMSC that exhibit variation in their ability to form heterotopic bone when implanted *in vivo* [21]. Our results demonstrate that there is phenotypic association between the *in vivo* bone formation and migratory capacity to bone fracture sites, and furthermore identified platelet-derived growth factor receptor (PDGFR)α and PDGFRβ as potential markers for the hBMSC population with enhanced migratory function.

## Methods

### Human mesenchymal stem cell culture

As a model for primary hBMSC, we employed our well-characterized telomerized hBMSC-TERT cell line, established by ectopic expression of the catalytic subunit of human telomerase as described previously [23]. The hBMSC-TERT cells exhibit a stable cellular and molecular phenotype during *in vitro* culture similar to that of primary hBMSC [24]. The derivation and characterization of hBMSC-TERT^+Bone^, hBMSC-TERT^–Bone^, and high bone-forming (HBF) and low bone-forming (LBF) single-cell clones have been described previously by our group [21]. In brief, the hBMSC-TERT^+Bone^ subpopulation was derived from early-passage hBMSC-TERT cells (population doubling level 77), and showed high capacity for *in vivo* heterotopic bone formation, while the hBMSC-TERT^–Bone^ subpopulation was derived from hBMSC-TERT cells (population doubling level 233), and showed LBF capacity. Both the HBF and LBF single-cell clones were derived from hBMSC-TERT^+Bone^ cells by the limiting dilution method.

The cells were cultured in a standard growth medium containing minimal essential medium (MEM) (Gibco, Invitrogen, Herlev, Denmark) supplemented with 10 % fetal calf serum (FCS) (Biochrom, Berlin, Germany) and 1 % penicillin/streptomycin (Gibco, Invitrogen, Herlev, Denmark) at 37 °C in a humidified atmosphere containing 5 % CO_2_. The medium was changed every third day until cells became 90 % confluent.

### Studying cell spreading and focal adhesion formations

Cell culture plates were coated with fibronectin (10 μg/ml) in phosphate-buffered saline (PBS) for 2 hours at 37 °C, rinsed twice with PBS, and blocked with 1 % bovine serum albumin (BSA) for 1 hour. Cells were trypsinized, washed twice with MEM, and resuspended in serum-free medium for 1 hour at room temperature. Cell were then replated onto fibronectin-coated plates in standard culture medium supplemented with platelet-derived growth factor (PDGF)-BB (100 ng/ml) for 30 minutes at 37 °C. Cells were fixed in 4 % paraformaldehyde for 10 minutes, washed with PBS, and stained for F-actin with Phalloidin–fluorescein isothiocyanate (FITC) (Sigma, Brøndby, Denmark) and for focal adhesion with mouse monoclonal anti-Vinculin antibody (Sigma, Brøndby, Denmark) using Alexa Fluor® 488-conjugated rabbit anti-mouse IgG (H + L) as secondary antibody (Cell Signaling). Fluorescent staining was visualized by the Operetta® High Content Imaging system (Perkin Elmer, Rodgau, Germany) at 20× magnification. Fluorescent images were analyzed using Harmony® High Content Imaging Analysis Software (Perkin Elmer, Rodgau, Germany).

### Microarray analysis

Microarray analysis was performed on hBMSC-TERT^+Bone^, hBMSC-TERT^–Bone^, and hBMSC-TERT-derived single-cell clones: HBF (three clones; DD8, AD10, BB10) and LBF (three clones; CF1, CB4, CB8). To perform global gene expression analysis, 500 ng quality-checked total RNA per sample in triplicate were amplified and labeled according to the manufacturer’s protocol (Illumina TotalPrep RNA Amplification Kit; Ambion, Austin, TX, USA [25]). The resulting biotinylated cRNA was purified and hybridized to Illumina HumanRef-8 v3 Expression BeadChips (Illumina, San Diego, CA, USA [26]) on the Illumina Beadstation 500 platform. This was followed by washing, blocking, staining with streptavidin-Cy3, and quantitative detection of the fluorescent image of the array as specified by the manufacturer.

Raw data were processed using the Gene Expression Module version 1.8.0 provided with the GenomeStudio software (Illumina). This included background subtraction and normalization according to the “rank invariant” algorithm. Genes were considered “expressed” if the corresponding “Detection P-Value” given by the GenomeStudio software was *p*_det_ <0.01. Differential gene expression analysis was computed using the Illumina Custom Model. *p* values of differentially expressed genes (“Diff P-Values”) were modified by the Benjamini and Hochberg false discovery rate (FDR) correction algorithm [27]. Genes were considered differentially expressed genes if: they exhibited twofold upregulation or downregulation in average signal intensity; the corresponding FDR-adjusted “Diff P-Value” was *p*_adj_ <0.05; or the gene of interest was expressed in at least one of the samples under consideration.

Differentially upregulated genes in group 1 (hBMSC-TERT^+Bone^ and three HBF clones) when compared with group 2 (hBMSC-TERT^–Bone^ and three LBF clones) are presented in Table 1.

**Table 1:** Genes differentially upregulated in HBF clones + hBMSC-TERT^+Bone^ versus LBF clones + hBMSC-TERT^–Bone^

Category	PROBE_ID	Gene symbol	Gene name	Fold change	*p* value	Gene function
Migration	ILMN_1798360	CXCR7	Chemokine (C-X-C motif) receptor 7	30.34323	1.57 × 10^–5^	Receptor for CXCL12/SDF1
	ILMN_1689111	CXCL12	Chemokine (C-X-C motif) ligand 12	7.64381	6.57 × 10^–5^	Activates the C-X-C chemokine receptor CXCR4 to induce a rapid and transient rise in the level of intracellular calcium ions and chemotaxis
	ILMN_2373791	ENPP2	Ectonucleotide pyrophosphatase/phosphodiesterase 2	6.481839	1.58 × 10^–13^	Leukocyte-endothelial cell adhesion
	ILMN_1722713	FBLN1	Fibulin 1	5.060076	8.1 × 10^–17^	Plays a role in cell adhesion and migration
	ILMN_2082585	SNAI2	Snail homolog 2	4.84552	3.28 × 10^–25^	Involved in the generation and migration of neural crest cells
	ILMN_1811313	SLIT3	Slit homolog 3	4.378596	1.14 × 10^–15^	Acts as molecular guidance cue in cellular migration
	ILMN_1802646	EPHB6	EPH receptor B6	4.171862	0.00022	Modulates cell adhesion and migration
	ILMN_2086470	PDGFRA	Platelet-derived growth factor receptor, alpha polypeptide	4.168592	0.009945	Receptor that binds both PDGFA and PDGFB
	ILMN_2339266	LAMA2	Laminin, alpha 2	3.959818	5.47 × 10^–6^	Mediates the attachment, migration, and organization of cells into tissues during embryonic development
	ILMN_2307903	VCAM1	Vascular cell adhesion molecule 1	3.080952	7.75 × 10^–5^	Plays a role in leukocyte endothelial cell adhesion
	ILMN_1757338	PLSCR4	Phospholipid scramblase 4	2.885336	0.008172	Mediates bidirectional transbilayer migration of phospholipids, plays a central role in the initiation of fibrin clot formation and activation of mast cells and in the recognition of apoptotic and injured cells
	ILMN_1761540	SEMA3F	Sema domain, immunoglobulin domain (Ig), short basic domain, secreted, (semaphorin) 3 F	2.657031	8.68 × 10^–8^	Plays a role in cell motility and cell adhesion
	ILMN_1659306	SVIL	Supervillin	2.569266	0.001237	May modulate myosin II regulation through MLCK during cell spreading, an initial step in cell migration
	ILMN_1667893	TNS3	Tensin 3	2.446932	5.1 × 10^–6^	Involved in cell migration and bone development
	ILMN_1815057	PDGFRB	Platelet-derived growth factor receptor	2.147921	5.33 × 10^–10^	Receptor that binds both PDGF-AA and PDGF-BB
Adhesion	ILMN_2408683	PPAP2B	Phosphatidic acid phosphatase type 2B	8.923443	4.45 × 10^–11^	May be involved in cell adhesion and in cell–cell interactions
	ILMN_1684554	COL16A1	Collagen, type XVI, alpha 1	3.998721	4.96 × 10^–12^	Involved in mediating cell attachment and induction of integrin-mediated cellular reactions, such as cell spreading and alterations in cell morphology
	ILMN_1801246	IFITM1	Interferon induced transmembrane protein 1 (9–27)	3.467308	1.36 × 10^–14^	Component of a complex involved in adhesion signals
	ILMN_2396444	CD14	CD14 molecule	3.169754	0.000265	Upregulates cell surface molecules, including adhesion molecules
	ILMN_2307903	VCAM1	Vascular cell adhesion molecule 1	3.080952	7.75 × 10^–5^	Plays a role in leukocyte endothelial cell adhesion
	ILMN_2229877	PCDH18	Protocadherin 18	2.939445	6.56 × 10^–8^	Potential calcium-dependent cell-adhesion protein
	ILMN_1761540	SEMA3F	Sema domain, immunoglobulin domain (Ig), short basic domain, secreted	2.657031	8.68 × 10^–8^	May play a role in cell motility and cell adhesion
	ILMN_1812461	WISP2	WNT1 inducible signaling pathway protein 2	2.336378	0.001181	Promotes the adhesion of osteoblast cells and inhibits the binding of fibrinogen to integrin receptors
	ILMN_1769575	JAM3	Junctional adhesion molecule 3	2.195171	4.39 × 10^–5^	May participate in cell–cell adhesion
	ILMN_2223941	FBLN5	Fibulin 5	2.099419	0.000119	Promotes adhesion of endothelial cells through interaction of integrins
	ILMN_2115125	CTGF	Connective tissue growth factor	2.037551	3 × 10^–9^	Mediates heparin-dependent and divalent cation-dependent cell adhesion
	ILMN_1707070	PCOLCE	Procollagen C-endopeptidase enhancer	2.023961	1.28 × 10^–5^	Procollagen C-endopeptidase enhancer
Bone formation	ILMN_1696391	LEPR	Leptin receptor	9.88173	0.000244	Leptin receptor
	ILMN_1690945	CPZ	Carboxypeptidase Z	6.636527	2.46 × 10^–11^	Modulates the Wnt signaling pathway
	ILMN_1800317	WNT5A	Wingless-type MMTV integration site family, member 5A	4.39779	5.65 × 10^–6^	Can activate or inhibit canonical Wnt signaling
	ILMN_1709734	BMP4	Bone morphogenetic protein 4	3.425185	0.003465	Induces cartilage and bone formation
	ILMN_1758895	CTSK	Cathepsin K (CTSK), mRNA.	2.655974	0.000136	Closely involved in osteoclastic bone resorption
	ILMN_1684755	KAZALD1	Kazal-type serine peptidase inhibitor domain 1	2.583283	0.001055	Involved in the proliferation of osteoblasts during bone formation and bone regeneration
	ILMN_1724480	AXIN2	Axin 2	2.432503	5.5 × 10^–7^	Downregulates beta-catenin
	ILMN_1770161	BST1	Bone marrow stromal cell antigen 1	2.417137	0.002254	Involved in osteoclastic bone resorption
	ILMN_1729368	FZD8	Frizzled homolog 8	2.013491	0.005335	Receptor for Wnt proteins
Cytoskeletal	ILMN_1812031	PALM	Paralemmin	9.661952	0.004851	Control of cell shape
	ILMN_1780334	KCNJ2	Potassium inwardly-rectifying channel, subfamily J, member 2	9.409812	1.23 × 10^–21^	Participates in establishing action potential waveform and excitability of neuronal and muscle tissues
	ILMN_1654319	HAPLN3	Hyaluronan and proteoglycan link protein 3	6.178261	3.95 × 10^–27^	May function in hyaluronic acid binding
	ILMN_1741695	COL12A1	Collagen, type XII, alpha 1	2.071144	0.008916	
	ILMN_1670899	FBN2	Fibrillin 2	2.065329	0.000762	Contains microfibrils that regulate the early process of elastic fiber assembly
	ILMN_1674620	SGCE	Sarcoglycan, epsilon	2.034948	7.35 × 10^–6^	A subcomplex of the dystrophin–glycoprotein complex which forms a link between the F-actin cytoskeleton and the extracellular matrix
	ILMN_1780334	KCNJ2	Potassium inwardly-rectifying channel, subfamily J, member 2	9.409812	1.23 × 10^–21^	Participates in establishing action potential waveform and excitability of neuronal and muscle tissues

*HBF* high bone forming, *hBMSC* human bone marrow-derived stromal cells, *LBF* low bone forming, *PDGF* platelet-derived growth factor

Functional annotation and identification of enriched biological categories were carried out using DAVID platform version 6.7 [28–30].

The microarray data have been deposited in the public repository [GEO:GSE69358].

### Total RNA isolation and real-time PCR

RNA was isolated from cultured cells using Trizol® (Bio-Rad, Herlev, Denmark) according to the manufacturer’s protocol. cDNA was synthesized using a commercial revertAid H minus first strand cDNA synthesis kit (Fermentas, Copenhagen, Denmark) according to the instruction manual. Real-time PCR was performed in the iCycler IQ detection system (Bio-Rad, Herlev, Denmark) using SYBR® Green I as described previously [24]. Primer sequences are indicated in Table S1 in Additional file 1. The expression level for each target gene was calculated using the comparative Ct formula 1/(2^∆Ct^) and data were presented as relative expression to the reference genes (RG) (HPRT1 and UBC). Data were analyzed using Microsoft Excel 2000 to generate relative expression values.

### Flow cytometry analysis

Cells were trypsinized and washed with PBS containing 0.5 % BSA (Fluorescence Activated Cell Sorting (FACS) buffer) and 50 μg/ml trypsin inhibitor. Cells were then incubated with primary antibody for PDGFRβ (0.025 mg/ml, PR292a; R&D Systems (Abingdon, UK) diluted 1:50 in FACS buffer for 30 minutes on ice. The cells were washed twice with ice-cold FACS buffer and incubated with FITC-conjugated goat anti-mouse antibody (R&D Systems) for 30 minutes on ice. The cells were analyzed by flow cytometer Cell Lab Quanta TMSC (Beckman Coulter using Kaluza version1.2 (Beckman Coulter, Copenhagen, Denmark).

### Magnetic activated cell sorting of hBMSC-TERT^–Bone^

Cells were rinsed with PBS, lifted with trypsin, and diluted in Magnetic activated cell sorting (MACS) buffer (PBS with 0.5 % (w/v) BSA and 2 mM ethylenediamine tetraacetic acid (EDTA)) before incubation with PDGFRβ antibody (R&D Systems) for 20 minutes at 4 °C. Cells were washed with MACS buffer, incubated with anti-mouse-beads (Miltenyi Biotec, Lund, Sweden) for 15 minutes at 4 °C, and run through a MS column (Miltenyi Biotec, Lund, Sweden) according to the manufacturer’s instructions.

### Generating the Luciferase-overexpressing hBMSC-TERT^+Bone^ and hBMSC-TERT^–Bone^

The hBMSC-TERT^+Bone^ and hBMSC-TERT^–Bone^ cells were transduced with a retroviral vector containing the firefly luciferase gene (Luc2) producing luciferase-containing hBMSC-TERT (hBMSC-TERT-Luc). The luciferase reporter gene luc2 (*Photinus pyralis*) was subcloned into the retroviral vector pBABE-puro (addgene Plasmid 1764). Retroviral vector was transfected into the Phoenix-AMPHO packaging cells (CRL-3213™; ATCC), and cultured in Dulbecco’s modified Eagle’s medium (DMEM) medium (Gibco, Invitrogen, Herlev, Denmark) supplemented with 10 % FCS until 70–80 % confluent, using the FuGENE 6 (Roche, Hvidovre, Denmark) reagent according to the manufacturer’s instruction. For cell transduction, the supernatants collected from Phoenix packaging cells containing virus particles were filtered with a 0.45 μm filter and added to hBMSC-TERT cell lines in the presence of Polybrene (Sigma, Brøndby, Denmark). Stably transduced cells were selected on the antibiotic puromycine.

### *In vitro* differentiation studies and cytochemical staining

#### Osteoblast differentiation

Cells were plated at 2 × 10^4^ cells/cm^2^ in six-well plates in modified Dulbecco medium (MEM) (GIBCO, Herlev, Denmark) containing 10 % fetal bovine serum (FBS) (GIBCO), 100 U/ml penicillin (GIBCO), and 100 μg/ml streptomycin (GIBCO). One day after plating, media were replaced with osteogenic media including culture media supplemented with 10 mM β-glycerophosphate (Sigma, Brøndby, Denmark), 10 nM dexamethasone (Sigma, Brøndby, Denmark), 10 nM Calcitriol (1,25-dihydroxyvitamin D3; Leo, Ballerup, Denmark), and 50 μg/ml vitamin C (Sigma, Brøndby, Denmark). The osteogenic medium was changed every other day for 2 weeks.

#### Adipocyte differentiation

For adipocyte differentiation, cells were plated at 3 × 10^4^ cells/cm^2^ in six-well plates in culture media. The day after, culture media was removed and adipogenic media was added including MEM (GIBCO), 10 % FBS (GIBCO), 10 % horse serum (GIBCO), 100 U/ml penicillin (GIBCO), 100 μg/ml streptomycin (GIBCO), 100 nM dexamethasone (Sigma, Brøndby, Denmark), 0.25 mM 3-isobutyl-1-methylxanthine (IBMX, Gibco, Herlev, Denmark), 1 μM BRL (Rosiglitazone; Sigma, Brøndby, Denmark), and 500 nM insulin (Sigma, Brøndby, Denmark,). The adipogenic medium was changed every other day for 20 days.

#### Alkaline phosphatase staining

At 7 and 14 days during osteoblast differentiation, cells were fixed with acetone/citrate (10 mM) buffer, pH 4.2 (1.5:1), for 5 minutes at room temperature and stained with the 1:1 mixture of 0.2 mg/ml Napthol-AS-TR-phosphate (Sigma) and 1.3 mg/ml Fast Red TR solution (Sigma) in 0.1 M Tris buffer, pH 9.0, for 1 hour at room temperature.

#### Alizarin Red staining

Alizarin Red staining was performed on days 7 and 14 of osteoblast differentiation to assess matrix mineralization of osteoblastic cultures. Cells were fixed with 70 % ice-cold ethanol for 1 hour at −20 °C, and stained with 40 mM Alizarin Red S (AR-S; Sigma, Brøndby, Denmark), pH 4.2, for 10 minutes at room temperature. The amount of mineralized matrix (bound stain) was quantified by eluting the Alizarin Red stain, using 20-minute incubation of the cultures in 10 % (w/vol) cetylpyridinium chloride solution. The absorbance of the eluted stain was measured at 570 nm using FLUOstar Omega multimode microplate reader (Leica, Ballerup, Denmark).

#### Oil Red O staining

Adipocytic cultures from days 10 and 20 were fixed with 4 % paraformaldehyde for 10 minutes at room temperature, rinsed with 3 % isopropanol solution, and stained with Oil Red O (Sigma, USA) solution for 1 hour at room temperature. Oil Red O solution consist of 25 mg Oil Red O dye, 5 ml of 60 % isopropanol, and 3.35 ml H_2_O. The bound dye was eluted by 100 % isopropanol and absorbance measured at 490 nm using FLUOstar Omega multimode microplate reader.

### Microscopy

Images were taken using a Leica® DM4500B microscope equipped with a motorized stage (Märzhäuser Wetzlar GmbH, Wetzlar, Germany) and a Leica® DFC300 FX camera. Images were acquired with the Surveyor® software (Objective Imaging, Leica, Ballerup, Denmark).

### Boyden chamber transwell migration assay

For migration experiments, 90 % confluent cells were starved for 24 hours in low-glucose Dulbecco’s minimal essential medium (LG-DMEM; GIBCO, Herlev, Denmark) supplemented with 0.1 % (w/v) BSA (Sigma, Brøndby, Denmark), harvested, and assayed using the AP48 Migration Assay system and 8 μm pore size polycarbonate membranes (NeuroProbe, Inc., USA). Membranes were coated for 1 hour at 37 °C with 5 μg/ml fibronectin (human placenta; Sigma) and 10 μg/ml rat tail collagen type I (Sigma, Brøndby, Denmark) in migration media consisting of LG-DMEM (GIBCO) supplemented with 0.2 % FBS (GIBCO). hBMSC-TERT cells in LG-DMEM with 0.1 % BSA (control medium) with or without pretreatment (see later) were added to the wells. Control media with or without PDGFs were added to the lower chamber. The assembled assay was incubated at 37 °C for 4 hours. After removal of cells on the upper side of the membrane with a rubber scraper, cell migration through the membrane was quantitated by staining cells with the Hemacolor staining kit (Merck), and cells were counted from one picture/well taken at 10× magnification by a computer-assisted cell-counting program (Visiopharm, Hørsholm, Denmark). The following concentrations of factors were used: PDGF-BB (PeproTech GmbH Hamburg, Germany), 10–100 ng/ml; PDGF-AA (PeproTech GmbH Hamburg, Germany), 100 ng/ml; PDGF-AB (PeproTech GmbH Hamburg, Germany), 100 ng/ml; and PDGFRβ inhibitor (SU16f; Tocris), 10–200 nM. Then 1 μl/ml vehicle (dimethyl sulfoxide (DMSO)) were added to the cells just prior to assay assembly.

### *In vivo* homing studies

#### Animals

Ethical permission for animal study was granted from the Danish National Authority on animal experiments (2012-15-2934-00559). Immunodeficient NOD scid (NOD.CB17-*Prkdc*^*scid*^*/J*) mice were used and operations were performed under intraperitoneal injection of ketamine (100 mg/kg) and xylazine (10 mg/kg). Temgesic (100 mg/kg) was given subcutaneously every 8 hours to relieve postoperative pain for at least 3 days post procedure.

### Closed femur fracture

The closed femur fracture procedure was carried out as described by Bonnarens and Einhorn [31]. In brief, an incision was made just medial to the patella, and then the longitudinal fibers of the quadriceps were divided and the patella dislocated laterally exposing the femur condyles. A 0.5 mm needle was inserted between the condyles and drilled in a retrograde fashion into the medullary cavity of the femur (functioning as a nail fixating the fracture). Afterwards, a standardized reproducible closed fracture was induced using a guillotine fracture apparatus [31]. A radiograph was acquired following fracture induction (using Faxitron MX-20; Faxitron X-Ray , Lincolnshire, USA) to confirm the fracture induction and needle position.

### Transplantation studies and bioluminescence imaging

Immunodeficient NOD/SCID (NOD/LtSz/*Prkdc*^*scid*^*/J*) 12-week-old female mice (*n* = 24) were randomly divided into the two main fracture study groups. The hBMSC-TERT^+Bone^ (*n* = 12) or hBMSC-TERT^–Bone^ cells (*n* = 12) were injected intravenously via tail vein at a dose of 1 × 10^6^ cells/mouse suspended in 200 μl PBS per injection. For noninvasive imaging analysis, mice were injected with d-Luciferin (150 mg/kg, intraperitoneal injection; Caliber Life Sciences), and after 10 minutes the mice were placed in a supine position for image acquisition with 5 minutes of acquisition time using the IVIS Imaging System, 200 Series (Xenogen Corp., Rodgau, Germany). Bioluminescence color images were overlaid on gray-scale photographic images of the mice to allow for localization of the light source using the Livingimage™ software (V. 2.11) overlay (Xenogen Corp., Rodgau, Germany). To equalize comparisons across animals and between groups, the scales were fixed. Regions of interest (ROI) were manually selected, and signal intensity was expressed in terms of number of photons/cm^2^/second.

### Statistical analysis

Data are presented as mean ± standard error of the mean SEM. Statistical analysis was performed using unpaired, two-tailed Student’s *t* test for comparison between two groups and one-way analysis of variance for comparison between more than two groups using Graphpad Prism version 5.0 software (Graphpad Prism Software, Inc., La Jolla, CA, USA). For all experiments, *p* <0.05 compared with control was considered significant.

## Results

### hBMSC-TERT^+Bone^ cells demonstrate high *ex vivo* directed migratory function

**Fig. 1:**
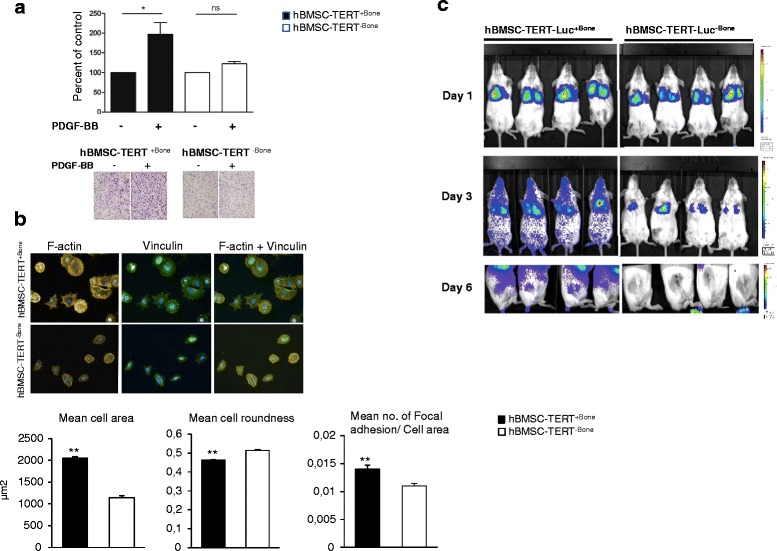
The capacity of hBMSC for ectopic bone formation is associated with enhancing *ex vivo* migration and *in vivo* homing to fractured bone. **a** Transwell migration ability of hBMSC-TERT^+Bone^ versus hBMSC-TERT^–Bone^ toward PDGF-BB (100 ng/ml). Migrated cells presented as percentage of control condition (without chemoattractant). Photomicrographs represent stained migrated cells. **b** Cytoskeletal changes in hBMSC-TERT^+Bone^ versus hBMSC-TERT^–Bone^ after plating on fibronectin-coated plates for 2 hours and stained for F-actin with Phalloidin–FITC (*yellow*) and vinculin (*green*). **c** Longitudinal imaging of representative mice that received intravenous injection of 1 × 10^6^ hBMSC-TERT-Luc^+Bone^ (*left panel*) or hBMSC-TERT-Luc^–Bone^ (*right panel*) cells. By day 6, signal from homing hBMSC-TERT-Luc^+Bone^ was detected at the right (fractured) leg while no signal was detected in the fractured legs of mice receiving hBMSC-TERT-Luc^–Bone^. Data presented as mean ± SEM of at least three independent experiments (*p ≤0.05, **p ≤0.01). *hBMSC* human bone marrow stromal stem cells, *PDGF* platelet-derived growth factor

To study whether the ability to form heterotopic bone *in vivo* is associated with an enhancement of the migratory capacity of hBMSC, we compared the *ex vivo* transwell migration ability of two populations derived from hBMSC-TERT cells: one capable of *in vivo* bone formation (hBMSC-TERT^+Bone^) versus one that lacks this ability (hBMSC-TERT^–Bone)^ [21]. As shown in Fig. 1a, the transwell migration ability of hBMSC-TERT^+Bone^ toward PDGF-BB was significantly higher compared with hBMSC-TERT^–Bone^.

### hBMSC-TERT^+Bone^ cells exhibit distinct cytoskeletal properties

Since cell migration includes a cascade of cellular events of cell spreading and focal adhesion formation [32], we compared cytoskeletal and adhesion properties of hBMSC-TERT^+Bone^ versus hBMSC-TERT^–Bone^ when cultured on fibronectin-coated plates for 2 hours and stained for F-actin and vinculin. As shown in Fig. 1b, hBMSC-TERT^+Bone^ exhibited more cells spreading with formation of cytoskeletal projections, increased mean cell area, and reduced mean cell roundness. In addition, the focal adhesion number per cell area, as visualized by vinculin staining, was increased in hBMSC-TERT^+Bone^ (Fig. 1b).

### hBMSC-TERT^+Bone^ cells exhibit enhanced *in vivo* homing to fractured bone site

We further compared the *in vivo* homing ability of hBMSC-TERT^+Bone^ versus hBMSC-TERT^–Bone^ to bone fracture sites in mice. hBMSC-TERT^+Bone^ and hBMSC-TERT^–Bone^ were stably transduced with a retroviral vector expressing the luciferase gene 2 (Luc2). Luc-overexpressing cell lines exhibited high levels of luciferase enzyme activity and maintained their differentiation capacity similar to the parental cell lines (Figure S1a, b in Additional file 1). hBMSC-TERT-Luc^+Bone^ and hBMSC-TERT-Luc^–Bone^ were administrated intravenously via tail vein injection in SCID/NOD mice with a stabilized femur bone fracture immediately following fracture induction. Noninvasive bioluminescence imaging using the IVIS Imaging System showed trapping of both cell lines in the lungs after 1 day (Fig. 1c). hBMSC-TERT^+Bone^ cells and not hBMSC-TERT^–Bone^, were able to migrate to the fracture sites by day 6 (Fig. 1c).

### Molecular phenotype of hBMSC single-cell clones with enhanced migratory function

**Fig. 2:**
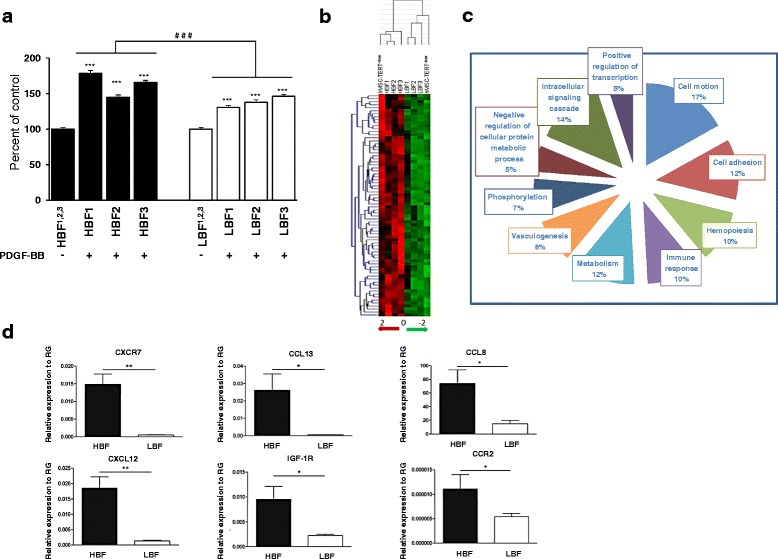
Transcriptional upregulation of the migratory-related factors by hBMSC with high bone formation capacity. **a** The transwell migration ability of three high bone-forming (HBF1, HBF2, HBF3) clones versus three low bone-forming (LBF1, LBF2, LBF3) clones toward PDGF-BB (100 ng/ml). Migrated cells presented as percentage of control condition (without chemoattractant). Data presented as mean ± SEM of at least three independent experiments per each clone (***p ≤0.005 as compared with nontreated control; ###*p* ≤0.005 between the mean value of three HBF clones versus the mean value of three LBF clones). **b** Heat map of molecular signature of hBMSC-TERT^+Bone,^ hBMSC-TERT^–Bone^, HBF clones (HBF1, HBF2, HBF3), and LBF clones (LBF1, LBF2, LBF3). Hierarchical clustering analysis based on Pearson’s correlation (group gene profile, overall expression data) between different groups shows the clustering of HBF clones with hBMSC-TERT^+Bone^ and LBF clones with hBMSC-TERT^–Bone^. **c** Annotation analysis of differentially upregulated genes (*p* <0.01, twofold cutoff) by HBF clones versus LBF clones according to gene molecular function. **d** Real-time RT-PCR analysis of some upregulated genes (from microarray analysis by HBF clones versus LBF cones). The expression of each target gene was normalized to RG and presented as relative expression to RG. Data presented as mean (three different clones of both HBF and LBF clones) ± SEM of at least three independent experiments (*p ≤0.05, **p ≤0.01, between LBF versus HBF clones). *PDGF* platelet-derived growth factor, *RG* reference genes

Since hBMSC-TERT^+Bone^ and hBMSC-TERT^–Bone^ are nonclonal populations, we further studied the migratory capacity of three hBMSC-TERT-derived single-cell clones with either HBF capacity (HBF1, HBF2, and HBF3) or three cell clones with LBF capacity (LBF1, LBF2, and LBF3) [21]. HBF clones showed higher migratory capacity toward PDGF-BB as compared with LBF clones, supporting our hypothesis of the existence of an association between the *in vivo* bone formation capacity and *ex vivo* migratory capacity of hBMSC (Fig. 2a).

To identify the molecular signature underlying enhanced hBMSC migratory function, we performed a global DNA microarray analysis comparing the transcriptome profile of hBMSC-TERT^+Bone^, hBMSC-TERT^–Bone^, the three HBF clones (HBF1, HBF2, and HBF3), and the three LBF clones (LBF1, LBF2, and LBF3). Cluster analysis revealed molecular clustering of HBF clones to hBMSC-TERT^+Bone^ and LBF clones to hBMSC-TERT^–Bone^ (Fig. 2b). We identified 427 genes (cutoff level ≥2-fold, *p* 0.05) that are upregulated in hBMSC-TERT^+Bone^ and HBF clones compared with hBMSC-TERT^–Bone^ and LBF clones (Table S2 in Additional file 1). Gene annotation analysis based on molecular functions revealed significant enrichment of gene categories of cell migration (12 %), cell adhesion (17 %), cytoskeletal genes (12 %), and bone-related genes (16 %) in hBMSC-TERT^+Bone^ and HBF clones (Fig. 2c, Table 1). Some of the significantly upregulated genes by HBF clones were confirmed by real-time RT-PCR (Fig. 2d).

### PDGFRα and PDGFRβ are associated with enhanced hBMSC migration

**Fig. 3:**
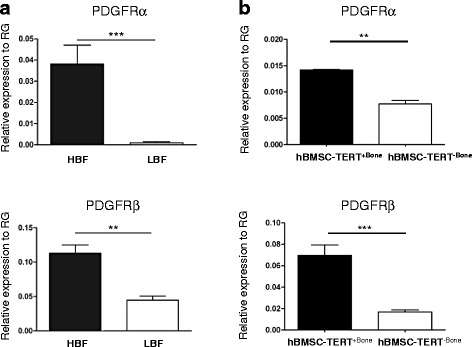
PDGFRα/β expression in HBF versus LBF. **a** Real-time RT-PCR analysis of PDGFRα and PDGFRβ expression by HBF versus LBF clones, and **b** hBMSC-TERT^+Bone^ versus hBMSC-TERT^–Bone^. Expression of each target gene was normalized to RG and presented as relative expression to RG. Data presented as mean (three different clones of each HBF and LBF clones) ± SEM of at least three independent experiments (*p ≤0.05, **p ≤0.01, ***p ≤0.005). *HBF* high bone forming, *hBMSC* human bone marrow stromal stem cells, *LBF* low bone forming, *PDGFR* platelet-derived growth factor receptor, *RG* reference genes

To identify surface marker(s) that can be used prospectively to isolate the hBMSC population with enhanced migratory functions, we examined differentially expressed genes in HBF clones. Both PDGFRα (CD140A) and PDGFRβ (CD140B) were found to be significantly upregulated by hBMSC-TERT^+Bone^ and HBF clones versus hBMSC-TERT^–Bone^ and LBF clones by 4.16-fold and 2.14-fold respectively (Table 1). These data have also been confirmed by quantitative RT-PCR analysis (Fig. 3a, b).

**Fig. 4:**
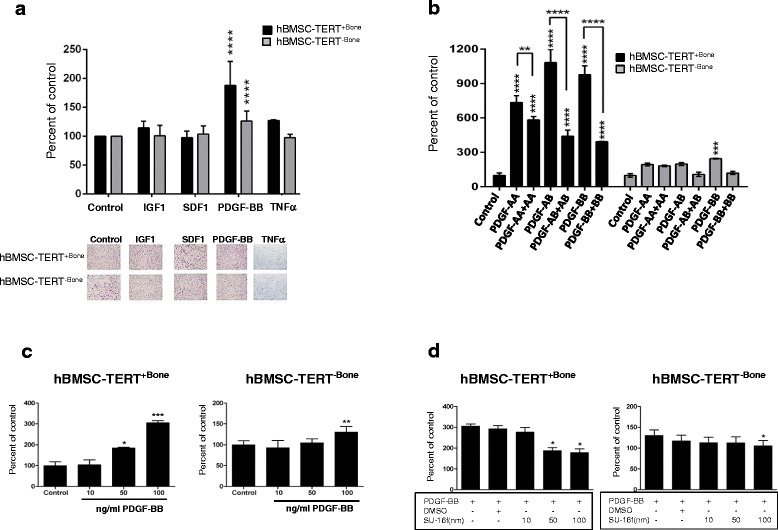
PDGF selectively enhanced the migration of hBMSC-TERT^+Bone^ versus hBMSC-TERT^–Bone^. **a** Transwell migration ability of hBMSC-TERT^+Bone^ versus hBMSC-TERT^–Bone^ toward the chemoattractants including: IGF-1 (10 ng/ml), SDF-1 (100 ng/ml), PDGF-BB (100 ng/ml), and TNFα (10 ng/ml). Photomicrographs represent images of the migrated cells for each condition. **b** Transwell migration assay of hBMSC-TERT^+Bone^ and hBMSC-TERT^–Bone^ cells toward different subtypes of PDGF recombinant protein (100 ng/ml). PDGF isoforms have applied either in the lower chamber (PDGF-AA, PDGF-AB, PDGF-BB) of the transwell migration assay to examine the migratory capacity of the cells or applied to both lower and upper chambers of the assay (PDGF-AA + PDGF-AA, PDGF-AB + PDGF-Ab, PDGF-BB + PDGF-BB) to inhibit the migration. **c** Dose-dependent effect of PDGF-BB (10, 50, or 100 ng/ml) on the transwell migration of hBMSC-TERT^+Bone^ or hBMSC-TERT^–Bone^ cells. **d** Dose-dependent effect of SU-16f inhibitor (a selective inhibitor for PDGFRβ) on the transwell migration of hBMSC-TERT^+Bone^ or hBMSC-TERT^–Bone^ cells. Data presented as mean ± SEM of at least three independent experiments (*p ≤0.05, **p ≤0.01, ***p ≤0.005, ****p ≤0.001, as compared with control). *DMSO* dimethyl sulfoxide, *hBMSC* human bone marrow stromal stem cells, *IGF1* insulin-like growth factor 1, *PDGF* platelet-derived growth factor, *SDF1*stromal cell-derived factor 1, *TNFα* tumor necrosis factor alpha

To test for the specific contribution of PDGFRs to migratory functions, we tested the effect of PDGF and other growth factors known to exert chemoattractant function: insulin-like growth factor 1 (IGF1), stromal cell-derived factor 1 (SDF1), and tumor necrosis factor alpha (TNFα). The *ex vivo* transwell migratory capacity of hBMSC-TERT^+Bone^ was significantly higher toward PDGF-BB compared with other chemoattractants (Fig. 4a). We next confirmed the specificity of chemotactic effects of PDGF isoforms on hBMSC-TERT^+Bone^. As shown in Fig. 4b, hBMSC-TERT^+Bone^ exhibited higher migration toward heterodimeric PDGF-AB or homodimeric PDGF-AA and PDGF-BB proteins and their migration was decreased by adding the same concentration of PDGF isoform to both lower and upper chambers in the transwell migration assay (Fig. 4b). Furthermore, we demonstrated a dose-dependent stimulatory effect of PDGF-BB on increasing the migration of hBMSC-TERT^+Bone^ versus hBMSC-TERT^–Bone^ (Fig. 4c). In addition, selective inhibition of PDGFRβ signaling in hBMSC-TERT^+Bone^ using the chemical inhibitor SU-16f suppressed the migration of hBMSC-TERT^+Bone^ toward PDGF-BB in a dose-dependent manner (Fig. 4d).

### Identifying PDGFRβ as a marker for prospective isolation of a population of hBMSC with migratory function

**Fig. 5:**
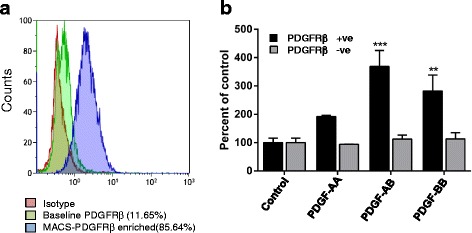
PDGFRβ as a potential marker for enhancing hBMSC migration. **a** FACS analysis of PDGFRβ in hBMSC-TERT^–Bone^ before and after enrichment for PDGFRβ^+^ cells. **b** MACS PDGFRβ^+^-enriched cells from hBMSC-TERT^–Bone^ were compared with PDGFRβ^–^ cells for their migratory response toward different PDGF isoforms (PDGF-AA, PDGF-BB, or PDGF-AB) (100 ng/ml). Data presented as mean ± SEM of at least three independent experiments (*p ≤0.05, **p ≤0.01, **p ≤0.005, as compared with control). *PDGFR* platelet-derived growth factor receptor

To study the possible use of PDGFRβ as a candidate marker for isolating hBMSC with increased migratory capacity, MACS-enriched PDGFRβ^+^ cells from hBMSC-TERT^–Bone^ (Fig. 5a) were obtained and the PDGFRβ^+^ cells were examined for their response to PDGF isoforms in the transwell migration assay. Sorted PDGFRβ^+^ cells exhibited enhanced chemotactic migration toward different PDGF isoforms as compared with the PDGFRβ-negative population (Fig. 5b).

## Discussion

In the present study, we demonstrate that a subpopulation of hBMSC with known ability to form bone *in vivo* exhibits enhanced ability to migrate *in vitro* and to home to bone fractures *in vivo*. In addition, we identified a molecular signature that defines this population and one surface marker, PDGFRβ (CD 140b), that can be employed for prospective isolation of the cells with enhanced migratory capacity from heterogeneous hBMSC cultures.

hBMSC are capable of multilineage differentiation into various mesoderm-type cells including osteoblastic cells [33], which is the basis for their use in cell-based therapies for bone tissue regeneration; for example, in repair of bone defects and nonhealed fractures [34–36]. However, cultured hBMSC represent a heterogeneous population with respect to differentiation capacity (so-called progenitor functions) as well as nonprogenitor functions. For example, not all hBMSC maintained under standard culture conditions are capable of bone formation [19]. Similarly, when hBMSC are infused intravenously, only a small population of cells home to injured tissue [37]. The efficient use of hBMSC in therapy thus requires defining an *ex vivo* molecular phenotype predictive for the *in vivo* behavior [21]. In the present study, we have investigated the correlation between two characteristics: cellular migratory behavior and ability to form heterotopic bone, as well as homing capacity to bone fractures *in vivo*. For this purpose, we employed previously characterized hBMSC clones with various capacities for *in vivo* heterotopic bone formation. Our data suggest that *in vivo* heterotopic bone formation capacity is associated with enhanced *in vivo* migratory abilities to bone fractures.

By comparing the transcriptome profile of hBMSC-TERT^+Bone^ and hBMSC-TERT^–Bone^ and clones with different heterotopic bone-forming capacities, we identified a molecular signature linking homing and *in vivo* bone formation potential. This molecular signature included expression of high levels of known genes important for cell migration and homing. Among these were chemokine (C-X-C motif) receptor 7 (CXCR7) and chemokine (C-X-C motif) ligand 12 (CXCL12) [38], extracellular matrix, and adhesion molecules (e.g., collagen, type XVI, alpha 1 (COL16A1) and vascular cell adhesion molecule 1 (VCAM-1)) [39].

We have demonstrated that hBMSC with bone-forming capacity can home to bone fracture, suggesting that these migratory cells are recruited from the circulation under physiological conditions of bone formation and bone regeneration. However, the contribution of homed hBMSC to bone regeneration at bone fracture sites has been a contentious issue [40]. Some studies have demonstrated that circulating hBMSC can home and contribute to fracture healing [36, 41, 42]. Also, patients with recent fractures have a large number of circulating osteoprogenitor (i.e., hBMSC-like) cells in their peripheral circulation, providing circumstantial evidence of recruiting bone-forming cells to bone fracture sites [43]. On the other hand, some studies have demonstrated that the contribution of homed hBMSC to bone fracture healing is limited and that fracture healing is mediated by locally recruited cells possibly from periosteum or bone marrow [44, 45]. Our study suggests that there exists a population of hBMSC with enhanced bone-forming and migratory capacity. Further prospective studies are needed to quantitate the contribution of this cell population to fracture healing and bone regeneration.

Some of the molecules identified in the molecular signature defining high bone formation and migratory capacity do not seem to contribute to the migratory phenotype. For example, CXCR7 expression was highly upregulated in cells with HBF capacity, but we were unable to detect an increased *ex vivo* chemotactic migration toward its ligand SDF-1. It is possible that other noncanonical receptors are involved in regulating the migratory function of hBMSC. It is also plausible that hBMSC differ from leucocytes regarding the molecules employed for transendothelial migration. In support of this notion, we did not detect increased homing of hBMSC to bone fracture *in vivo* in hBMSC overexpressing CXCR4 (data not shown), a known factor enhancing leukocyte and HSC homing [46].

We identified high expression levels of PDGFRα and PDGFRβ to be associated with enhanced chemotactic migration of hBMSC toward PDGF isoforms. Therefore, PDGFRβ can be used as a possible marker to enrich for hBMSC with high *ex vivo* migratory capacity. PDGFs, via binding to their cognate receptors PDGFRα and PDGFRβ, have been reported previously as potent chemoattractants for multiple cell types including hBMSC [47–50]. Our study corroborates and extends previous findings by showing the possible prospective isolation of hBMSC with enhanced migratory capacity based on PDGFRβ expression.

Our study has some limitations. First, we employed a telomerized cell line (hBMSC-TERT) and not primary hBMSC for isolating subpopulations of BMSC. hBMSC-TERT has the advantage of having a stable phenotype during long-term culture and our extensive experience with hBMSC-TERT demonstrate that these cells maintain a phenotype similar to primary hBMSC *in vitro* and *in vivo* [24] and has comparable molecular signature to primary hBMSC [51]. While hMSC-TERT has been employed in a clinical trial as a vehicle for drug delivery [52], the cells are not suitable for clinical trials for bone regeneration due to their genetic modification and the risk of transformation. Future studies on the relevance of our findings to *in vivo* bone regeneration should thus be conducted using primary human cells in preclinical animal models [53]. Second, the identified PDGFRβ can be both a marker and functional protein contributing to enhance migration. Our studies do not distinguish between these two possibilities. Third, prospective isolation of PDGFRβ^+^ cells from heterogeneous hBMSC cultures that can both home and contribute to fracture healing needs to be performed.

## Conclusion

This study demonstrates that cultured hBMSC, in addition to cellular heterogeneity, exhibit functional heterogeneity and that bone formation and homing abilities are closely linked characteristics that can be defined by a common molecular signature. Further studies are needed to determine the role of PDGFRβ in hBMSC homing and to isolate hBMSC populations based on their molecular signature to be tested in preclinical animal models for enhancing fracture healing and bone regeneration.

## Additional file

Additional file 1:
**Figure S1**. Showing generation and differentiation of hBMSC-TERT-Luc cell lines, **Table S1** presenting a list of primers used for RT-PCR, and **Table S2** presenting genes upregulated by group 1 (hMSC-TERT^+Bone^ and three HBF clones) over group 2 (hMSC-TERT^–Bone^ and three LBF clones).(PDF 317 kb)

## Abbreviations

BMSC: Bone marrow-derived stromal cells
BSA: Bovine serum albumin
DMSO: Dimethyl sulfoxide
EDTA: Ethylenediamine tetraacetic acid
FBS: Fetal bovine serum
FCS: Fetal calf serum
FDR: False discovery rate
FITC: Fluorescein isothiocyanate
HBF: High bone forming
hBMSC: Human bone marrow-derived stromal stem cells
HSC: Hematopoietic stem cell
IGF-1: Insulin-like growth factor 1
LBF: Low bone forming
LG-DMEM: Low-glucose Dulbecco’s minimal essential medium
MEM: Minimal essential medium
PBS: Phosphate-buffered saline
PDGF: Platelet-derived growth factor
PDGFR: Platelet-derived growth factor receptor
RG: Reference genes
ROI: Regions of interest
SDF1: Stromal cell derived factor 1
SEM: Standard error of the mean
TNFα: Tumor necrosis factor alpha
