# Supplementary material for: Association between in vivo bone formation and ex vivo migratory capacity of human bone marrow stromal cells
Source: Stem Cell Res Ther. 2015 Oct 8;6:196. doi: 10.1186/s13287-015-0188-9 (PMC4599318; doi:10.1186/s13287-015-0188-9)
Supplement: Additional file 1: — Figure S1. Showing generation and differentiation of hBMSC-TERT-Luc cell lines, Table S1 presenting a list of primers used for RT-PCR, and Table S2 presenting genes upregulated by group 1 (hMSC-TERT+Bone and three HBF clones) over group 2 (hMSC-TERT–Bone and three LBF clones).(PDF 317 kb) [file 13287_2015_188_MOESM1_ESM.pdf]

Figure S1

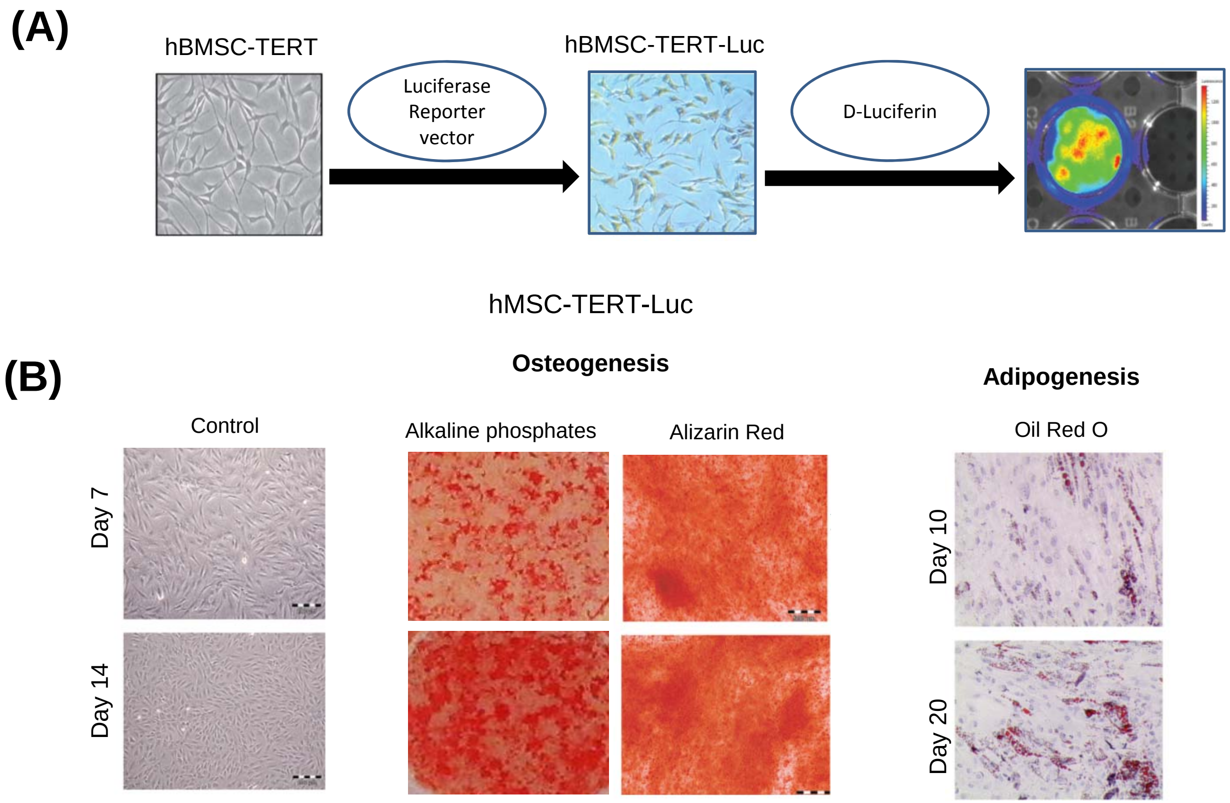

Supplementary figure 1: Generation and differntiation of hBMSC-TERT-Luc cell lines
(A) The hBMSC-TERT^{+Bone} and hBMSC-TERT^{-Bone} cells were transduced with an adenoviral vector containing the firefly luciferase gene (Luc2) producing luciferase-containing hBMSCs (hBMSC-TERT-Luc).
(B) Luc-overexpressing cells were induced either to differentiate into osteoblast or adipocyte lineages. Osteoblast differentiation was assessed by Alkaline phosphatase and Alizarin Red staining for matrix mineralization, while adipocyte differentiation was assessed by Oild Red O staining for lipid droplets.

Table S1. List of primers used for qRT-PCR.

Gene symbol	Forward primer 5´-3´	Reverse Primer 5´-3´
CCL13	ACATGAAAGTCTCTGCAGTGCTTC	AGTAGATGGGACGTTGAGTGCAT
CCL2	TGCTGTACCAAGAGTTTGCTC	CGCACACAGACAACTTTTTCTTT
C-Kit	TCATGGTCGGATCACAAAGA	AGGGGCTGCTTCCTAAAGAG
CXCR4	CTGAGAAGCATGACGGACAAGTACAG	CAACAGCTTCCTTGGCCTCTGACT
CXCR7	AGCACAGCCAGGAAGGCGAG	TCATAGCCTGTGGTCTTGGC
CD82	GCCGACAAGAGCAGTTTCAT	GGAAGCCCATGAGCATAGTG
CDH1	AGAACGCATTGCCACATACACTC	CATTCTGATCGGTTACCGTGATC
IL10	ATGCCCCAAGCTGAGAACCAAGAC	TCTCAAGGGGCTGGGTCAGCTATC
IL1beta	AGGAAGATGCTGGTTCCCTGC	CAGTTCAGTGATCGTACAGGTGC
IL8	ATGACTTCCAAGCTGGCCGTG	TTATGAATTCTCAGCCCTCTTCAAAAACTT
IGF1R	CTAAACCCGGGGAACTACACAG	TTCACAGAGGCATACAGCAC
PDGFRα	GAGATCCACTCCCGAGACAG	AGCAGCCACCGTGAGTTC
PDGFRβ	AAT GTC TCC AGC ACC TTC GT	AGC GGA TGT GGT AAG GCA TA
HPRT1	TGACACTGGCAAAACAATGCA	GGTCCTTTTCACCAGCAAGCT
UBC	ATTTGGGTCGCGGTTCTTG	TGCCTTGACATTCTCGATGGT

Table S2: Genes upregulated by group 1 (hMSC-TERT^{+Bone} and 3 HBF clones) over group 2 (hMSC-TERT^{-Bone} and 3 LBF clones).

Gene Symbol	Gene Name	Fold change	P val
STK32B	serine/threonine kinase 32B (STK32B), mRNA.	37,69537203	< 0,05
CXCR7	chemokine (C-X-C motif) receptor 7 (CXCR7), mRNA.	28,27461115	< 0,0005
PTGES	prostaglandin E synthase (PTGES), mRNA.	17,6109191	< 0,0005
MAN1C1	mannosidase, alpha, class 1C, member 1 (MAN1C1), mRNA.	15,55089143	< 0,05
ELN	elastin (ELN), transcript variant 4, mRNA.	15,3061288	< 0,05
CRISPLD2	cysteine-rich secretory protein LCCL domain containing 2 (CRISPLD2), mRNA.	13,70372165	< 0,00005
SLC2A12	solute carrier family 2 (facilitated glucose transporter), member 12 (SLC2A12), mRNA.	13,62400419	< 0,05
CH25H	cholesterol 25-hydroxylase (CH25H), mRNA.	13,08092051	< 0,05
NOTCH3	Notch homolog 3 (Drosophila) (NOTCH3), mRNA.	12,85114985	< 0,0005
OLFML2B	olfactomedin-like 2B (OLFML2B), mRNA.	9,785572254	< 0,0005
C10ORF116	chromosome 10 open reading frame 116 (C10orf116), mRNA.	9,628352634	< 0,00005
SMOC1	SPARC related modular calcium binding 1 (SMOC1), transcript variant 1, mRNA.	9,54945956	< 0,05
PALM	paralemmin (PALM), transcript variant 1, mRNA.	9,372041735	< 0,0005
LEPR	leptin receptor (LEPR), transcript variant 1, mRNA.	9,369949978	< 0,00005
DBC1	deleted in bladder cancer 1 (DBC1), mRNA.	9,307140612	< 0,00005
KCNJ2	potassium inwardly-rectifying channel, subfamily J, member 2 (KCNJ2), mRNA.	9,065295896	< 0,0005
PPAP2B	phosphatidic acid phosphatase type 2B (PPAP2B), transcript variant 2, mRNA.	9,014786691	< 0,00005
MXRA5	matrix-remodelling associated 5 (MXRA5), mRNA.	8,881111723	< 0,05
PAPPA	pregnancy-associated plasma protein A, pappalysin 1 (PAPPA), mRNA.	8,858756892	< 0,00005
AKR1B10	aldo-keto reductase family 1, member B10 (aldose reductase) (AKR1B10), mRNA.	8,721129093	< 0,05
SCRG1	scrapie responsive protein 1 (SCRG1), mRNA.	8,62782389	< 0,05
FCGRT	Fc fragment of IgG, receptor, transporter, alpha (FCGRT), mRNA.	8,484467012	< 0,00005
TXNIP	thioredoxin interacting protein (TXNIP), mRNA.	8,427442068	< 0,00005
COX7A1	cytochrome c oxidase subunit VIIa polypeptide 1 (muscle) (COX7A1), mRNA.	8,372060423	< 0,05
FMO3	flavin containing monooxygenase 3 (FMO3), transcript variant 1, mRNA.	8,097040697	< 0,0005
VMO1	vitelline membrane outer layer 1 homolog (chicken) (VMO1), mRNA.	8,002540187	< 0,05
DAAM2	dishevelled associated activator of morphogenesis 2 (DAAM2), mRNA.	7,903706919	< 0,0005
CXCL12	chemokine (C-X-C motif) ligand 12 (stromal cell-derived factor 1) (CXCL12), transcript variant 2, mRNA.	7,726119293	< 0,00005

AGT	angiotensinogen (serpin peptidase inhibitor, clade A, member 8) (AGT), mRNA.	7,573678592	< 0,05
SCUBE3	signal peptide, CUB domain, EGF-like 3 (SCUBE3), mRNA.	7,517575698	< 0,05
JAG1	jagged 1 (Alagille syndrome) (JAG1), mRNA.	7,272863141	< 0,00005
C11ORF87	chromosome 11 open reading frame 87 (C11orf87), mRNA.	6,871431887	< 0,00005
OLFML1	olfactomedin-like 1 (OLFML1), mRNA.	6,840018518	< 0,0005
CD248	CD248 molecule, endosialin (CD248), mRNA.	6,552910584	< 0,00005
CPZ	carboxypeptidase Z (CPZ), transcript variant 1, mRNA.	6,531392639	< 0,0005
FOXQ1	forkhead box Q1 (FOXQ1), mRNA.	6,509257815	< 0,05
SVEP1	sushi, von Willebrand factor type A, EGF and pentraxin domain containing 1 (SVEP1), mRNA.	6,500128207	< 0,00005
ENPP2	ectonucleotide pyrophosphatase/phosphodiesterase 2 (ENPP2), transcript variant 2, mRNA.	6,383561223	< 0,00005
C13ORF33	chromosome 13 open reading frame 33 (C13orf33), mRNA.	6,347162844	< 0,00005
PDE4D	phosphodiesterase 4D, cAMP-specific (phosphodiesterase E3 dunce homolog, Drosophila) (PDE4D), mRNA.	6,237206638	< 0,0005
HAPLN3	hyaluronan and proteoglycan link protein 3 (HAPLN3), mRNA.	6,133587066	< 0,00005
SIX2	SIX homeobox 2 (SIX2), mRNA.	6,11465129	< 0,05
SOX18	SRY (sex determining region Y)-box 18 (SOX18), mRNA.	5,948026566	< 0,05
MAP1A	microtubule-associated protein 1A (MAP1A), mRNA.	5,913337355	< 0,00005
FAM43A	family with sequence similarity 43, member A (FAM43A), mRNA.	5,717098092	< 0,05
TGFB3	transforming growth factor, beta 3 (TGFB3), mRNA.	5,703525592	< 0,05
C1QTNF5	C1q and tumor necrosis factor related protein 5 (C1QTNF5), mRNA.	5,377892217	< 0,0005
MFAP4	microfibrillar-associated protein 4 (MFAP4), mRNA.	5,313771066	< 0,05
PODN	podocan (PODN), mRNA.	5,309027352	< 0,00005
MDK	midkine (neurite growth-promoting factor 2) (MDK), transcript variant 1, mRNA.	5,233743546	< 0,05
FBLN1	fibulin 1 (FBLN1), transcript variant A, mRNA.	5,124224005	< 0,0005
IL8	interleukin 8 (IL8), mRNA.	5,113806398	< 0,00005
PLEKHG4	pleckstrin homology domain containing, family G (with RhoGef domain) member 4 (PLEKHG4), mRNA.	5,067595008	< 0,0005
EDNRA	endothelin receptor type A (EDNRA), mRNA.	5,047852008	< 0,05
CKB	creatine kinase, brain (CKB), mRNA.	5,039939269	< 0,00005
PDE7B	phosphodiesterase 7B (PDE7B), mRNA.	5,027007311	< 0,00005

PRRX2	paired related homeobox 2 (PRRX2), mRNA.	4,959366171	< 0,00005
ALDH3A2	aldehyde dehydrogenase 3 family, member A2 (ALDH3A2), transcript variant 1, mRNA.	4,896953691	< 0,0005
SNAI2	snail homolog 2 (Drosophila) (SNAI2), mRNA.	4,879917118	< 0,00005
FMO2	flavin containing monooxygenase 2 (non-functional) (FMO2), mRNA.	4,877293815	< 0,05
COL18A1	collagen, type XVIII, alpha 1 (COL18A1), transcript variant 2, mRNA.	4,823791642	< 0,05
HEY2	hairy/enhancer-of-split related with YRPW motif 2 (HEY2), mRNA.	4,751220021	< 0,05
ISG20	interferon stimulated exonuclease gene 20kDa (ISG20), mRNA.	4,637293847	< 0,00005
MAF	v-maf musculoaponeurotic fibrosarcoma oncogene homolog (avian) (MAF), transcript variant 2, mRNA.	4,634200376	< 0,05
PIR	pirin (iron-binding nuclear protein) (PIR), transcript variant 2, mRNA.	4,502226289	< 0,0005
KYNU	kynureninase (L-kynurenine hydrolase) (KYNU), transcript variant 2, mRNA.	4,476555409	< 0,0005
ZNF423	zinc finger protein 423 (ZNF423), mRNA.	4,468637982	< 0,0005
WNT5A	wingless-type MMTV integration site family, member 5A (WNT5A), mRNA.	4,434877385	< 0,00005
EPHX1	epoxide hydrolase 1, microsomal (xenobiotic) (EPHX1), mRNA.	4,409365174	< 0,00005
PPP1R3C	protein phosphatase 1, regulatory (inhibitor) subunit 3C (PPP1R3C), mRNA.	4,404345074	< 0,00005
PLAC9	placenta-specific 9 (PLAC9), mRNA.	4,385480159	< 0,00005
SLC47A1	solute carrier family 47, member 1 (SLC47A1), mRNA.	4,380759198	< 0,0005
S100A4	S100 calcium binding protein A4 (S100A4), transcript variant 2, mRNA.	4,370571392	< 0,0005
SLIT3	slit homolog 3 (Drosophila) (SLIT3), mRNA.	4,365079809	< 0,00005
RAB7B	RAB7B, member RAS oncogene family (RAB7B), mRNA.	4,35247272	< 0,0005
LRFN4	leucine rich repeat and fibronectin type III domain containing 4 (LRFN4), mRNA.	4,284916314	< 0,05
SLC4A11	solute carrier family 4, sodium borate transporter, member 11 (SLC4A11), mRNA.	4,198563463	< 0,05
SOCS2	suppressor of cytokine signaling 2 (SOCS2), mRNA.	4,19795629	< 0,00005
TNFSF15	tumor necrosis factor (ligand) superfamily, member 15 (TNFSF15), mRNA.	4,194570077	< 0,05
WISP1	WNT1 inducible signaling pathway protein 1 (WISP1), transcript variant 1, mRNA.	4,191939573	< 0,05
PDGFRA	platelet-derived growth factor receptor, alpha polypeptide (PDGFRA), mRNA.	4,181751813	< 0,00005
RGMA	RGM domain family, member A (RGMA), mRNA.	4,150158449	< 0,0005
GCLM	glutamate-cysteine ligase, modifier subunit (GCLM), mRNA.	4,10832346	< 0,00005

SETBP1	SET binding protein 1 (SETBP1), mRNA.	4,07372647	< 0,0005
GAS1	growth arrest-specific 1 (GAS1), mRNA.	4,068576227	< 0,00005
HECW2	HECT, C2 and WW domain containing E3 ubiquitin protein ligase 2 (HECW2), mRNA.	4,043448397	< 0,00005
STAT4	signal transducer and activator of transcription 4 (STAT4), mRNA.	4,02696673	< 0,0005
GREM2	gremlin 2, cysteine knot superfamily, homolog (Xenopus laevis) (GREM2), mRNA.	4,026768195	< 0,0005
HSPB7	heat shock 27kDa protein family, member 7 (cardiovascular) (HSPB7), mRNA.	4,006640581	< 0,00005
COL16A1	collagen, type XVI, alpha 1 (COL16A1), mRNA.	4,00624962	< 0,00005
EPHB6	EPH receptor B6 (EPHB6), mRNA.	3,988039452	< 0,0005
MGC26718	similar to ankyrin repeat domain 20A (MGC26718), mRNA.	3,957120957	< 0,0005
POPDC3	popeye domain containing 3 (POPDC3), mRNA.	3,927374328	< 0,0005
FST	follistatin (FST), transcript variant FST317, mRNA.	3,901547341	< 0,0005
LOXL3	lysyl oxidase-like 3 (LOXL3), mRNA.	3,870884096	< 0,00005
SMAD6	SMAD family member 6 (SMAD6), mRNA.	3,852357724	< 0,00005
KLHL3	kelch-like 3 (Drosophila) (KLHL3), mRNA.	3,834644664	< 0,0005
SUSD2	sushi domain containing 2 (SUSD2), mRNA.	3,820105584	< 0,0005
IGFBP4	insulin-like growth factor binding protein 4 (IGFBP4), mRNA.	3,816593401	< 0,00005
GCHFR	GTP cyclohydrolase I feedback regulator (GCHFR), mRNA.	3,774305575	< 0,0005
HOXD12	homeobox D12 (HOXD12), mRNA.	3,761313871	< 0,00005
CRIP1	cysteine-rich protein 1 (intestinal) (CRIP1), mRNA.	3,758731257	< 0,00005
GHDC	GH3 domain containing (GHDC), mRNA.	3,756682751	< 0,0005
C16ORF45	chromosome 16 open reading frame 45 (C16orf45), mRNA.	3,727582438	< 0,00005
ANKRD20A1	ankyrin repeat domain 20 family, member A1 (ANKRD20A1), mRNA.	3,721176722	< 0,0005
STON1	stonin 1 (STON1), mRNA.	3,684646376	< 0,05
LDB2	LIM domain binding 2 (LDB2), mRNA.	3,682172574	< 0,00005
PRKCH	protein kinase C, eta (PRKCH), mRNA.	3,6679004	< 0,00005
C14ORF139	PREDICTED: chromosome 14 open reading frame 139 (C14orf139), misc RNA.	3,635539866	< 0,05
GPNMB	glycoprotein (transmembrane) nmb (GPNMB), transcript variant 1, mRNA.	3,615054795	< 0,00005
C20ORF103	chromosome 20 open reading frame 103 (C20orf103), mRNA.	3,597346003	< 0,0005
RIMS3	regulating synaptic membrane exocytosis 3 (RIMS3), mRNA.	3,585835057	< 0,0005
AKR1C2	aldo-keto reductase family 1, member C2 (dihydrodiol dehydrogenase 2; bile acid binding protein; 3-alpha hydroxysteroid dehydrogenase, type III) (AKR1C2), transcript variant 1, mRNA. XM_943424 XM_943425 XM_943427	3,560057171	< 0,00005

GSTM1	glutathione S-transferase M1 (GSTM1), transcript variant 1, mRNA.	3,537303576	< 0,0005
LAMA2	laminin, alpha 2 (LAMA2), transcript variant 1, mRNA.	3,535382667	< 0,0005
IL15	interleukin 15 (IL15), transcript variant 3, mRNA.	3,517865965	< 0,00005
SPSB2	splA/ryanodine receptor domain and SOCS box containing 2 (SPSB2), mRNA.	3,514086451	< 0,0005
IFITM1	interferon induced transmembrane protein 1 (9-27) (IFITM1), mRNA.	3,500308648	< 0,00005
CETP	cholesteryl ester transfer protein, plasma (CETP), mRNA.	3,483379043	< 0,05
TNFRSF14	tumor necrosis factor receptor superfamily, member 14 (herpesvirus entry mediator) (TNFRSF14), mRNA.	3,475107306	< 0,00005
GSTM2	glutathione S-transferase M2 (muscle) (GSTM2), mRNA.	3,473582526	< 0,00005
SLC43A2	solute carrier family 43, member 2 (SLC43A2), mRNA.	3,464063497	< 0,05
PPAP2A	phosphatidic acid phosphatase type 2A (PPAP2A), transcript variant 1, mRNA.	3,419136318	< 0,00005
SERPING1	serpin peptidase inhibitor, clade G (C1 inhibitor), member 1 (SERPING1), transcript variant 2, mRNA.	3,418773316	< 0,0005
ANGPTL2	angiopoietin-like 2 (ANGPTL2), mRNA.	3,409487371	< 0,00005
BMP4	bone morphogenetic protein 4 (BMP4), transcript variant 1, mRNA.	3,395567617	< 0,0005
GFPT2	glutamine-fructose-6-phosphate transaminase 2 (GFPT2), mRNA.	3,367680919	< 0,0005
PLA2G4B	phospholipase A2, group IVB (cytosolic) (PLA2G4B), mRNA.	3,348776565	< 0,0005
CYP1B1	cytochrome P450, family 1, subfamily B, polypeptide 1 (CYP1B1), mRNA.	3,335083081	< 0,00005
KLHDC9	kelch domain containing 9 (KLHDC9), transcript variant 2, mRNA.	3,327514092	< 0,0005
TRIM47	tripartite motif-containing 47 (TRIM47), mRNA.	3,317133064	< 0,00005
EBF1	early B-cell factor 1 (EBF1), mRNA.	3,267828857	< 0,00005
ADCY4	adenylate cyclase 4 (ADCY4), mRNA.	3,262917238	< 0,00005
SASH1	SAM and SH3 domain containing 1 (SASH1), mRNA.	3,255267107	< 0,00005
SLC9A9	solute carrier family 9 (sodium/hydrogen exchanger), member 9 (SLC9A9), mRNA.	3,253632443	< 0,05
JAK2	Janus kinase 2 (a protein tyrosine kinase) (JAK2), mRNA.	3,242029405	< 0,0005
RFTN2	raftlin family member 2 (RFTN2), mRNA.	3,214295072	< 0,0005
ZMYND15	zinc finger, MYND-type containing 15 (ZMYND15), mRNA.	3,167814141	< 0,0005
SELENBP1	selenium binding protein 1 (SELENBP1), mRNA.	3,157355778	< 0,00005
SOD3	superoxide dismutase 3, extracellular (SOD3), mRNA.	3,155027517	< 0,0005

NQO1	NAD(P)H dehydrogenase, quinone 1 (NQO1), transcript variant 1, mRNA.	3,152090022	< 0,0005
GNG11	guanine nucleotide binding protein (G protein), gamma 11 (GNG11), mRNA.	3,149690753	< 0,00005
MRGPRF	MAS-related GPR, member F (MRGPRF), mRNA.	3,146970097	< 0,00005
GSTM4	glutathione S-transferase M4 (GSTM4), transcript variant 2, mRNA.	3,136938202	< 0,0005
CD14	CD14 molecule (CD14), transcript variant 2, mRNA.	3,126242953	< 0,0005
BMP6	bone morphogenetic protein 6 (BMP6), mRNA.	3,125933018	< 0,05
VCAM1	vascular cell adhesion molecule 1 (VCAM1), transcript variant 1, mRNA.	3,121150207	< 0,00005
GDPD5	glycerophosphodiester phosphodiesterase domain containing 5 (GDPD5), mRNA.	3,106693144	< 0,0005
SIPA1L2	signal-induced proliferation-associated 1 like 2 (SIPA1L2), mRNA.	3,101370984	< 0,05
RUNX3	runt-related transcription factor 3 (RUNX3), transcript variant 2, mRNA.	3,092078858	< 0,05
C5ORF23	chromosome 5 open reading frame 23 (C5orf23), mRNA.	3,090455231	< 0,00005
CLEC3B	C-type lectin domain family 3, member B (CLEC3B), mRNA.	3,083050296	< 0,00005
RGS11	regulator of G-protein signaling 11 (RGS11), transcript variant 1, mRNA.	3,080431396	< 0,05
TCEA3	transcription elongation factor A (SII), 3 (TCEA3), mRNA.	3,077048292	< 0,0005
C15ORF41	chromosome 15 open reading frame 41 (C15orf41), mRNA.	3,076965133	< 0,00005
LPPR4	plasticity related gene 1 (LPPR4), mRNA.	3,067891798	< 0,0005
C15ORF59	chromosome 15 open reading frame 59 (C15orf59), mRNA.	3,056545203	< 0,05
LPCAT4	lysophosphatidylcholine acyltransferase 4 (LPCAT4), mRNA.	3,048966238	< 0,0005
PTX3	pentraxin-related gene, rapidly induced by IL-1 beta (PTX3), mRNA.	3,034627916	< 0,05
PMP22	peripheral myelin protein 22 (PMP22), transcript variant 2, mRNA.	3,010047153	< 0,00005
ADRA2A	adrenergic, alpha-2A-, receptor (ADRA2A), mRNA.	3,009848392	< 0,05
GALM	galactose mutarotase (aldose 1-epimerase) (GALM), mRNA.	2,998624425	< 0,0005
CYGB	cytoglobin (CYGB), mRNA.	2,989576409	< 0,05
PRRT2	proline-rich transmembrane protein 2 (PRRT2), mRNA.	2,966855841	< 0,0005
PCDH18	protocadherin 18 (PCDH18), mRNA.	2,962163453	< 0,00005
ADCY9	adenylate cyclase 9 (ADCY9), mRNA.	2,961586865	< 0,00005
ASAP3	ArfGAP with SH3 domain, ankyrin repeat and PH domain 3 1 (ASAP3), mRNA.	2,950745424	< 0,00005
AKR1C3	aldo-keto reductase family 1, member C3 (3-alpha	2,944162702	< 0,00005

	hydroxysteroid dehydrogenase, type II) (AKR1C3), mRNA.		
NOV	nephroblastoma overexpressed gene (NOV), mRNA.	2,943695074	< 0,00005
ATP8B4	ATPase, class I, type 8B, member 4 (ATP8B4), mRNA.	2,921777313	< 0,05
C5	complement component 5 (C5), mRNA.	2,91899585	< 0,00005
TRIB2	tribbles homolog 2 (Drosophila) (TRIB2), mRNA.	2,913795991	< 0,00005
TMEM130	transmembrane protein 130 (TMEM130), mRNA.	2,906601726	< 0,0005
PLSCR4	phospholipid scramblase 4 (PLSCR4), mRNA.	2,895234197	< 0,00005
IRS1	insulin receptor substrate 1 (IRS1), mRNA.	2,893269692	< 0,0005
ACACB	acetyl-Coenzyme A carboxylase beta (ACACB), mRNA.	2,888298201	< 0,0005
NME5	non-metastatic cells 5, protein expressed in (nucleoside-diphosphate kinase) (NME5), mRNA.	2,865854521	< 0,05
KIT	v-kit Hardy-Zuckerman 4 feline sarcoma viral oncogene homolog (KIT), mRNA.	2,856650214	< 0,05
CUL4B	cullin 4B (CUL4B), transcript variant 2, mRNA.	2,833062284	< 0,00005
FOXC1	forkhead box C1 (FOXC1), mRNA.	2,830165749	< 0,00005
CBLN3	cerebellin 3 precursor (CBLN3), mRNA.	2,814628907	< 0,00005
RARRES1	retinoic acid receptor responder (tazarotene induced) 1 (RARRES1), transcript variant 1, mRNA.	2,797423501	< 0,05
CLDN23	claudin 23 (CLDN23), mRNA.	2,795112096	< 0,0005
TMEM140	transmembrane protein 140 (TMEM140), mRNA.	2,794021804	< 0,00005
CYBA	cytochrome b-245, alpha polypeptide (CYBA), mRNA.	2,786140218	< 0,00005
CASC4	cancer susceptibility candidate 4 (CASC4), transcript variant 2, mRNA.	2,76537571	< 0,00005
DCN	decorin (DCN), transcript variant C, mRNA.	2,728208035	< 0,00005
PLCD1	phospholipase C, delta 1 (PLCD1), mRNA.	2,716819119	< 0,00005
FSIP1	fibrous sheath interacting protein 1 (FSIP1), mRNA.	2,716098608	< 0,05
LGMN	legumain (LGMN), transcript variant 2, mRNA.	2,716041455	< 0,0005
IVD	isovaleryl Coenzyme A dehydrogenase (IVD), nuclear gene encoding mitochondrial protein, mRNA.	2,706860698	< 0,00005
HES1	hairy and enhancer of split 1, (Drosophila) (HES1), mRNA.	2,702813517	< 0,05
RABGAP1	RAB GTPase activating protein 1 (RABGAP1), mRNA.	2,700932637	< 0,00005
ATOH8	atonal homolog 8 (Drosophila) (ATOH8), mRNA.	2,687194639	< 0,00005
SLC27A3	solute carrier family 27 (fatty acid transporter), member 3 (SLC27A3), mRNA.	2,681195807	< 0,0005
DLX5	distal-less homeobox 5 (DLX5), mRNA.	2,681012316	< 0,00005
PYCARD	PYD and CARD domain containing (PYCARD), transcript variant 1, mRNA.	2,680256443	< 0,05
SLC40A1	solute carrier family 40 (iron-regulated	2,672141921	< 0,05

	transporter), member 1 (SLC40A1), mRNA.		
SEMA3F	sema domain, immunoglobulin domain (Ig), short basic domain, secreted, (semaphorin) 3F (SEMA3F), mRNA.	2,665022462	< 0,00005
C21ORF63	chromosome 21 open reading frame 63 (C21orf63), mRNA.	2,653813907	< 0,05
ECGF1	endothelial cell growth factor 1 (platelet-derived) (ECGF1), mRNA.	2,651037737	< 0,00005
ICAM2	intercellular adhesion molecule 2 (ICAM2), transcript variant 1, mRNA.	2,643545649	< 0,0005
LRIG3	leucine-rich repeats and immunoglobulin-like domains 3 (LRIG3), mRNA.	2,64241659	< 0,05
EPAS1	endothelial PAS domain protein 1 (EPAS1), mRNA.	2,640849188	< 0,00005
ESPNL	espin-like (ESPNL), mRNA.	2,639888782	< 0,05
HOM-TES-103	hypothetical protein LOC25900, isoform 3 (HOM-TES-103), transcript variant 3, mRNA.	2,637877673	< 0,05
ZCCHC24	zinc finger, CCHC domain containing 24 (ZCCHC24), mRNA.	2,635505633	< 0,00005
SPRY4	sprouty homolog 4 (Drosophila) (SPRY4), mRNA.	2,629497556	< 0,0005
AMT	aminomethyltransferase (AMT), mRNA.	2,625183637	< 0,00005
CNKSR3	CNKSR family member 3 (CNKSR3), mRNA.	2,620220946	< 0,00005
CTSK	cathepsin K (CTSK), mRNA.	2,619329626	< 0,00005
AIFM2	apoptosis-inducing factor, mitochondrion-associated, 2 (AIFM2), nuclear gene encoding mitochondrial protein, mRNA.	2,616383705	< 0,0005
EPDR1	ependymin related protein 1 (zebrafish) (EPDR1), mRNA.	2,616161957	< 0,00005
IFFO1	intermediate filament family orphan 1 (IFFO1), transcript variant 2, mRNA.	2,612502266	< 0,0005
TMEM200A	transmembrane protein 200A (TMEM200A), mRNA.	2,60571892	< 0,00005
ZNF558	zinc finger protein 558 (ZNF558), mRNA.	2,604309553	< 0,05
CRIPAK	cysteine-rich PAK1 inhibitor (CRIPAK), mRNA.	2,603594407	< 0,00005
NPR3	natriuretic peptide receptor C/guanylate cyclase C (atrionatriuretic peptide receptor C) (NPR3), mRNA.	2,592515722	< 0,00005
MDFIC	MyoD family inhibitor domain containing (MDFIC), mRNA.	2,591472033	< 0,00005
APBB3	amyloid beta (A4) precursor protein-binding, family B, member 3 (APBB3), transcript variant 3, mRNA.	2,591346527	< 0,00005
KAZALD1	Kazal-type serine peptidase inhibitor domain 1 (KAZALD1), mRNA.	2,590258511	< 0,00005
AHR	aryl hydrocarbon receptor (AHR), mRNA.	2,572363842	< 0,00005
SVIL	supervillin (SVIL), transcript variant 2, mRNA.	2,568122306	< 0,00005
HSPB2	heat shock 27kDa protein 2 (HSPB2), mRNA.	2,567357874	< 0,00005
PITPNC1	phosphatidylinositol transfer protein, cytoplasmic 1 (PITPNC1), transcript variant 2, mRNA.	2,556041429	< 0,0005

CHST14	carbohydrate (N-acetylgalactosamine 4-0) sulfotransferase 14 (CHST14), mRNA.	2,547235277	< 0,00005
REV3L	REV3-like, catalytic subunit of DNA polymerase zeta (yeast) (REV3L), mRNA.	2,54552148	< 0,0005
C7ORF63	chromosome 7 open reading frame 63 (C7orf63), mRNA.	2,532971259	< 0,00005
FHOD1	formin homology 2 domain containing 1 (FHOD1), mRNA.	2,532594087	< 0,00005
CCDC32	coiled-coil domain containing 32 (CCDC32), transcript variant 2, mRNA.	2,529253208	< 0,00005
C9ORF150	chromosome 9 open reading frame 150 (C9orf150), mRNA.	2,526889565	< 0,0005
SYNGR1	synaptogyrin 1 (SYNGR1), transcript variant 1b, mRNA.	2,519405304	< 0,05
IFI30	interferon, gamma-inducible protein 30 (IFI30), mRNA.	2,510781499	< 0,05
DUSP10	dual specificity phosphatase 10 (DUSP10), transcript variant 2, mRNA.	2,510208613	< 0,05
ZNF471	zinc finger protein 471 (ZNF471), mRNA.	2,506680875	< 0,0005
LOC729021	PREDICTED: hypothetical protein LOC729021 (LOC729021), mRNA.	2,492626602	< 0,0005
C10ORF47	chromosome 10 open reading frame 47 (C10orf47), mRNA.	2,484777604	< 0,0005
TP73L	tumor protein p73-like (TP73L), mRNA.	2,481280443	< 0,0005
TNS3	tensin 3 (TNS3), mRNA.	2,475915302	< 0,00005
RCAN2	regulator of calcineurin 2 (RCAN2), mRNA.	2,473200656	< 0,00005
CPT1A	carnitine palmitoyltransferase 1A (liver) (CPT1A), nuclear gene encoding mitochondrial protein, transcript variant 2, mRNA.	2,464125193	< 0,05
STAG3L2	stromal antigen 3-like 2 (STAG3L2), mRNA.	2,457910026	< 0,00005
ANKRD29	ankyrin repeat domain 29 (ANKRD29), mRNA.	2,457684942	< 0,0005
C16ORF86	chromosome 16 open reading frame 86 (C16orf86), mRNA.	2,455586374	< 0,05
ANGPT1	angiopoietin 1 (ANGPT1), mRNA.	2,444130542	< 0,00005
C1R	complement component 1, r subcomponent (C1R), mRNA.	2,438322101	< 0,00005
VPS39	vacuolar protein sorting 39 homolog (S. cerevisiae) (VPS39), mRNA.	2,435886095	< 0,00005
KCNK2	potassium channel, subfamily K, member 2 (KCNK2), transcript variant 1, mRNA.	2,432812863	< 0,05
PLCD4	phospholipase C, delta 4 (PLCD4), mRNA.	2,429449488	< 0,05
ANTXR1	anthrax toxin receptor 1 (ANTXR1), transcript variant 1, mRNA.	2,428628065	< 0,00005
AXIN2	axin 2 (conductin, axil) (AXIN2), mRNA.	2,427976015	< 0,00005
LOC338799	hypothetical locus LOC338799 (LOC338799), non-coding RNA.	2,425794027	< 0,0005
PTGER4	prostaglandin E receptor 4 (subtype EP4) (PTGER4), mRNA.	2,425274205	< 0,0005

TMEM85	transmembrane protein 85 (TMEM85), mRNA.	2,424115014	< 0,00005
BST1	bone marrow stromal cell antigen 1 (BST1), mRNA.	2,423904713	< 0,00005
HCST	hematopoietic cell signal transducer (HCST), transcript variant 2, mRNA.	2,422627962	< 0,05
TTLL3	tubulin tyrosine ligase-like family, member 3 (TTLL3), transcript variant 2, mRNA.	2,416782524	< 0,0005
GALNTL1	UDP-N-acetyl-alpha-D-galactosamine:polypeptide N-acetylgalactosaminyltransferase-like 1 (GALNTL1), mRNA.	2,414609164	< 0,00005
RTN4RL1	reticulon 4 receptor-like 1 (RTN4RL1), mRNA.	2,403545235	< 0,05
ICA1L	islet cell autoantigen 1,69kDa-like (ICA1L), transcript variant 1, mRNA.	2,395977739	< 0,0005
TP53BP1	tumor protein p53 binding protein 1 (TP53BP1), mRNA.	2,39416873	< 0,00005
FAM110B	family with sequence similarity 110, member B (FAM110B), mRNA.	2,393307067	< 0,00005
SCAMP5	secretory carrier membrane protein 5 (SCAMP5), mRNA.	2,390134234	< 0,05
C1ORF158	chromosome 1 open reading frame 158 (C1orf158), mRNA.	2,386228524	< 0,05
MXRA8	matrix-remodelling associated 8 (MXRA8), mRNA.	2,386036602	< 0,00005
ZNF10	zinc finger protein 10 (ZNF10), mRNA.	2,380864148	< 0,05
ABCC3	ATP-binding cassette, sub-family C (CFTR/MRP), member 3 (ABCC3), mRNA.	2,378402794	< 0,00005
LPHN1	latrophilin 1 (LPHN1), transcript variant 2, mRNA.	2,378327576	< 0,0005
PDIA5	protein disulfide isomerase family A, member 5 (PDIA5), mRNA.	2,376363826	< 0,00005
KLF13	Kruppel-like factor 13 (KLF13), mRNA.	2,375380777	< 0,00005
KLHL13	kelch-like 13 (Drosophila) (KLHL13), mRNA.	2,37410702	< 0,05
RHOD	ras homolog gene family, member D (RHOD), mRNA.	2,369769453	< 0,00005
DLX1	distal-less homeobox 1 (DLX1), transcript variant 2, mRNA.	2,368097233	< 0,0005
FAM83H	family with sequence similarity 83, member H (FAM83H), mRNA.	2,364981806	< 0,0005
CRYAB	crystallin, alpha B (CRYAB), mRNA.	2,356596533	< 0,00005
ENO3	enolase 3 (beta, muscle) (ENO3), transcript variant 1, mRNA.	2,354642538	< 0,0005
C4ORF31	chromosome 4 open reading frame 31 (C4orf31), mRNA.	2,346890824	< 0,00005
GMDS	GDP-mannose 4,6-dehydratase (GMDS), mRNA.	2,34560491	< 0,00005
THBS1	thrombospondin 1 (THBS1), mRNA.	2,34468137	< 0,00005
PRRX1	paired related homeobox 1 (PRRX1), transcript variant pmx-1a, mRNA.	2,338193782	< 0,00005
CCPG1	cell cycle progression 1 (CCPG1), transcript variant 2, mRNA.	2,336850838	< 0,0005
PSD3	pleckstrin and Sec7 domain containing 3 (PSD3),	2,33290158	< 0,0005

	transcript variant 1, mRNA.		
LOH3CR2A	loss of heterozygosity, 3, chromosomal region 2, gene A (LOH3CR2A), mRNA.	2,321640953	< 0,05
FHDC1	FH2 domain containing 1 (FHDC1), mRNA.	2,319353658	< 0,00005
ZFP36	zinc finger protein 36, C3H type, homolog (mouse) (ZFP36), mRNA.	2,316849426	< 0,00005
LEPREL1	leprecan-like 1 (LEPREL1), mRNA.	2,316841625	< 0,00005
CAPS	calcyphosine (CAPS), transcript variant 1, mRNA.	2,315547271	< 0,0005
B3GALNT1	beta-1,3-N-acetylgalactosaminyltransferase 1 (globoside blood group) (B3GALNT1), transcript variant 2, mRNA.	2,310902095	< 0,0005
IFT140	intraflagellar transport 140 homolog (Chlamydomonas) (IFT140), mRNA.	2,30586862	< 0,00005
GULP1	GULP, engulfment adaptor PTB domain containing 1 (GULP1), mRNA.	2,304462535	< 0,0005
ADHFE1	alcohol dehydrogenase, iron containing, 1 (ADHFE1), transcript variant 1, mRNA.	2,299895592	< 0,0005
C3ORF15	chromosome 3 open reading frame 15 (C3orf15), mRNA.	2,297749322	< 0,0005
MTMR15	myotubularin related protein 15 (MTMR15), mRNA.	2,296605434	< 0,0005
HOXA5	homeobox A5 (HOXA5), mRNA.	2,29534992	< 0,00005
WISP2	WNT1 inducible signaling pathway protein 2 (WISP2), mRNA.	2,289897903	< 0,0005
MFGE8	milk fat globule-EGF factor 8 protein (MFGE8), mRNA.	2,286125609	< 0,00005
C10ORF33	chromosome 10 open reading frame 33 (C10orf33), mRNA.	2,283636537	< 0,00005
SNX21	sorting nexin family member 21 (SNX21), transcript variant 3, mRNA.	2,28146514	< 0,05
NAALADL1	N-acetylated alpha-linked acidic dipeptidase-like 1 (NAALADL1), mRNA.	2,269064281	< 0,05
SLC25A27	solute carrier family 25, member 27 (SLC25A27), nuclear gene encoding mitochondrial protein, mRNA.	2,268376205	< 0,0005
LOC440157	hypothetical gene supported by AK096951; BC066547 (LOC440157), mRNA.	2,263809858	< 0,00005
CYP26B1	cytochrome P450, family 26, subfamily B, polypeptide 1 (CYP26B1), mRNA.	2,261782276	< 0,0005
ADRB2	adrenergic, beta-2-, receptor, surface (ADRB2), mRNA.	2,258001846	< 0,05
CENTD3	centaurin, delta 3 (CENTD3), mRNA.	2,256308285	< 0,0005
C8ORF31	chromosome 8 open reading frame 31 (C8orf31), mRNA.	2,254008751	< 0,05
ST3GAL5	ST3 beta-galactoside alpha-2,3-sialyltransferase 5 (ST3GAL5), transcript variant 2, mRNA.	2,241515403	< 0,0005
FLJ45337	FLJ45337 protein (FLJ45337), mRNA.	2,238456936	< 0,05
PRR6	proline rich 6 (PRR6), mRNA.	2,237732678	< 0,00005

SERP2	stress-associated endoplasmic reticulum protein family member 2 (SERP2), mRNA.	2,237320244	< 0,0005
ITGB2	integrin, beta 2 (complement component 3 receptor 3 and 4 subunit) (ITGB2), mRNA.	2,229164685	< 0,00005
ID3	inhibitor of DNA binding 3, dominant negative helix-loop-helix protein (ID3), mRNA.	2,225264848	< 0,00005
FBXO6	F-box protein 6 (FBXO6), mRNA.	2,224588154	< 0,0005
ZNF502	zinc finger protein 502 (ZNF502), mRNA.	2,224291006	< 0,05
A4GALT	alpha 1,4-galactosyltransferase (A4GALT), mRNA.	2,222842055	< 0,00005
TFPI	tissue factor pathway inhibitor (lipoprotein-associated coagulation inhibitor) (TFPI), transcript variant 1, mRNA.	2,22184764	< 0,05
JAM3	junctional adhesion molecule 3 (JAM3), mRNA.	2,221361536	< 0,00005
KDELC2	KDEL (Lys-Asp-Glu-Leu) containing 2 (KDELC2), mRNA.	2,219000113	< 0,00005
ID1	inhibitor of DNA binding 1, dominant negative helix-loop-helix protein (ID1), transcript variant 2, mRNA.	2,215756813	< 0,00005
SLC6A6	solute carrier family 6 (neurotransmitter transporter, taurine), member 6 (SLC6A6), transcript variant 1, mRNA.	2,214939885	< 0,05
MKX	mohawk homeobox (MKX), mRNA.	2,214711039	< 0,00005
FAM131A	family with sequence similarity 131, member A (FAM131A), mRNA.	2,214469198	< 0,00005
LCMT2	leucine carboxyl methyltransferase 2 (LCMT2), mRNA.	2,209598461	< 0,00005
KIAA1407	KIAA1407 (KIAA1407), mRNA.	2,204397407	< 0,0005
PLD1	phospholipase D1, phosphatidylcholine-specific (PLD1), mRNA.	2,203902576	< 0,05
SPIRE2	spire homolog 2 (Drosophila) (SPIRE2), mRNA.	2,202593568	< 0,05
UBR1	ubiquitin protein ligase E3 component n-recognin 1 (UBR1), mRNA.	2,199294451	< 0,05
SNCAIP	synuclein, alpha interacting protein (SNCAIP), mRNA.	2,195400106	< 0,05
LMF1	lipase maturation factor 1 (LMF1), mRNA.	2,194405539	< 0,0005
DEPDC6	DEP domain containing 6 (DEPDC6), mRNA.	2,193049852	< 0,00005
SPRED1	sprouty-related, EVH1 domain containing 1 (SPRED1), mRNA.	2,191842377	< 0,00005
CSAD	cysteine sulfinic acid decarboxylase (CSAD), mRNA.	2,188754075	< 0,00005
GPR64	G protein-coupled receptor 64 (GPR64), transcript variant 4, mRNA.	2,18768312	< 0,0005
MSX1	msh homeobox 1 (MSX1), mRNA.	2,184466228	< 0,00005
ID2	inhibitor of DNA binding 2, dominant negative helix-loop-helix protein (ID2), mRNA.	2,179417109	< 0,00005
FAM46A	family with sequence similarity 46, member A (FAM46A), mRNA.	2,178586267	< 0,00005
ZFYVE19	zinc finger, FYVE domain containing 19 (ZFYVE19),	2,177215057	< 0,00005

	mRNA.		
TMEM87A	transmembrane protein 87A (TMEM87A), mRNA.	2,176739191	< 0,00005
PDGFRB	platelet-derived growth factor receptor, beta polypeptide (PDGFRB), mRNA.	2,176389301	< 0,00005
RBMS1	RNA binding motif, single stranded interacting protein 1 (RBMS1), transcript variant 2, mRNA.	2,17379453	< 0,05
WASF3	WAS protein family, member 3 (WASF3), mRNA.	2,168565618	< 0,00005
CTSL1	cathepsin L1 (CTSL1), transcript variant 1, mRNA.	2,168380799	< 0,00005
ZNF30	zinc finger protein 30 (ZNF30), transcript variant 2, mRNA.	2,163860622	< 0,05
ZIC4	Zic family member 4 (ZIC4), mRNA.	2,163401818	< 0,0005
LOC652968	hypothetical protein LOC652968 (LOC652968), mRNA.	2,163051291	< 0,00005
BCHE	butyrylcholinesterase (BCHE), mRNA.	2,157839327	< 0,0005
NPEPL1	aminopeptidase-like 1 (NPEPL1), mRNA.	2,154402892	< 0,0005
LMCD1	LIM and cysteine-rich domains 1 (LMCD1), mRNA.	2,146683436	< 0,00005
KIAA1751	KIAA1751 (KIAA1751), mRNA.	2,143787493	< 0,00005
KCNE4	potassium voltage-gated channel, Isk-related family, member 4 (KCNE4), mRNA.	2,143660649	< 0,05
ALDH5A1	aldehyde dehydrogenase 5 family, member A1 (ALDH5A1), nuclear gene encoding mitochondrial protein, transcript variant 2, mRNA.	2,138133716	< 0,0005
PTGR1	prostaglandin reductase 1 (PTGR1), mRNA.	2,137217589	< 0,00005
SULT1A4	sulfotransferase family, cytosolic, 1A, phenol-preferring, member 4 (SULT1A4), transcript variant 3, mRNA.	2,136939502	< 0,00005
NTN4	netrin 4 (NTN4), mRNA.	2,134824627	< 0,00005
H3F3B	H3 histone, family 3B (H3.3B) (H3F3B), mRNA.	2,130743931	< 0,00005
LMTK3	PREDICTED: lemur tyrosine kinase 3 (LMTK3), mRNA.	2,130543796	< 0,05
SSPN	sarcospan (Kras oncogene-associated gene) (SSPN), mRNA.	2,130102848	< 0,05
KLF4	Kruppel-like factor 4 (gut) (KLF4), mRNA.	2,128671142	< 0,00005
FLJ10357	hypothetical protein FLJ10357 (FLJ10357), mRNA.	2,127421564	< 0,0005
PGD	phosphogluconate dehydrogenase (PGD), mRNA.	2,126064761	< 0,00005
RTP4	receptor (chemosensory) transporter protein 4 (RTP4), mRNA.	2,124197464	< 0,05
FAM176B	family with sequence similarity 176, member B (FAM176B), mRNA.	2,118836986	< 0,00005
MX2	myxovirus (influenza virus) resistance 2 (mouse) (MX2), mRNA.	2,116666621	< 0,00005
C6ORF154	chromosome 6 open reading frame 154 (C6orf154), mRNA.	2,112841566	< 0,0005
PHF15	PHD finger protein 15 (PHF15), mRNA.	2,105744127	< 0,00005
YPEL3	yippee-like 3 (Drosophila) (YPEL3), mRNA.	2,104579931	< 0,00005
CCDC74A	coiled-coil domain containing 74A (CCDC74A), mRNA.	2,102901794	< 0,00005

LOC387856	similar to expressed sequence AI836003 (LOC387856), mRNA.	2,102060267	< 0,0005
SEMA3B	sema domain, immunoglobulin domain (Ig), short basic domain, secreted, (semaphorin) 3B (SEMA3B), transcript variant 2, mRNA.	2,101103109	< 0,0005
COL12A1	collagen, type XII, alpha 1 (COL12A1), transcript variant long, mRNA.	2,096588593	< 0,0005
TBX2	T-box 2 (TBX2), mRNA.	2,09533989	< 0,0005
LAMB3	laminin, beta 3 (LAMB3), transcript variant 1, mRNA.	2,09515352	< 0,05
FBLN5	fibulin 5 (FBLN5), mRNA.	2,094836651	< 0,00005
TSHZ1	teashirt zinc finger homeobox 1 (TSHZ1), mRNA.	2,093916267	< 0,00005
IGF1R	insulin-like growth factor 1 receptor (IGF1R), mRNA.	2,092447412	< 0,00005
SLC38A2	solute carrier family 38, member 2 (SLC38A2), mRNA.	2,089817297	< 0,00005
C15ORF57	chromosome 15 open reading frame 57 (C15orf57), transcript variant 3, mRNA.	2,087587048	< 0,05
UNC5B	unc-5 homolog B (C. elegans) (UNC5B), mRNA.	2,086341637	< 0,0005
RPL23AP13	ribosomal protein L23a pseudogene 13 (RPL23AP13), non-coding RNA.	2,085336192	< 0,0005
C8ORF45	chromosome 8 open reading frame 45 (C8orf45), mRNA.	2,084325549	< 0,00005
CAPN3	calpain 3, (p94) (CAPN3), transcript variant 3, mRNA.	2,084171091	< 0,05
PCBD2	pterin-4 alpha-carbinolamine dehydratase/dimerization cofactor of hepatocyte nuclear factor 1 alpha (TCF1) 2 (PCBD2), mRNA.	2,083923386	< 0,05
CYP4V2	cytochrome P450, family 4, subfamily V, polypeptide 2 (CYP4V2), mRNA.	2,083709606	< 0,00005
RALGPS2	Ral GEF with PH domain and SH3 binding motif 2 (RALGPS2), transcript variant 1, mRNA.	2,080874419	< 0,05
STAT1	signal transducer and activator of transcription 1, 91kDa (STAT1), transcript variant alpha, mRNA.	2,079641398	< 0,00005
MAP2K3	mitogen-activated protein kinase kinase 3 (MAP2K3), transcript variant A, mRNA.	2,079489345	< 0,0005
C15ORF24	chromosome 15 open reading frame 24 (C15orf24), mRNA.	2,078548242	< 0,00005
TNFAIP2	tumor necrosis factor, alpha-induced protein 2 (TNFAIP2), mRNA.	2,077221043	< 0,0005
FBN2	fibrillin 2 (congenital contractural arachnodactyly) (FBN2), mRNA.	2,076412376	< 0,00005
DCLK1	doublecortin-like kinase 1 (DCLK1), mRNA.	2,074697135	< 0,0005
ZNF177	zinc finger protein 177 (ZNF177), mRNA.	2,073168445	< 0,0005
LOC90586	AOC3 pseudogene (LOC90586), non-coding RNA.	2,072221647	< 0,00005
C1ORF176	chromosome 1 open reading frame 176 (C1orf176), mRNA.	2,069878385	< 0,0005
LGALS3	lectin, galactoside-binding, soluble, 3 (galectin 3)	2,069416233	< 0,05

	(LGALS3), mRNA.		
CALHM2	calcium homeostasis modulator 2 (CALHM2), mRNA.	2,068007213	< 0,0005
CTGF	connective tissue growth factor (CTGF), mRNA.	2,066324156	< 0,00005
IFNGR1	interferon gamma receptor 1 (IFNGR1), mRNA.	2,065760915	< 0,00005
ZMYND12	zinc finger, MYND-type containing 12 (ZMYND12), mRNA.	2,064871866	< 0,05
SGCE	sarcoglycan, epsilon (SGCE), transcript variant 3, mRNA.	2,059504729	< 0,00005
TMEM91	transmembrane protein 91 (TMEM91), mRNA.	2,058542566	< 0,00005
ATHL1	ATH1, acid trehalase-like 1 (yeast) (ATHL1), mRNA.	2,052779294	< 0,00005
PCOLCE	procollagen C-endopeptidase enhancer (PCOLCE), mRNA.	2,051401623	< 0,00005
C6ORF70	chromosome 6 open reading frame 70 (C6orf70), mRNA.	2,050790067	< 0,00005
RIPK4	receptor-interacting serine-threonine kinase 4 (RIPK4), mRNA.	2,049089531	< 0,0005
KIAA1026	kazrin (KIAA1026), transcript variant N, mRNA.	2,047320623	< 0,05
PCYOX1	prenylcysteine oxidase 1 (PCYOX1), mRNA.	2,047065123	< 0,00005
USP49	ubiquitin specific peptidase 49 (USP49), mRNA.	2,044484182	< 0,00005
C21ORF24	chromosome 21 open reading frame 24 (C21orf24), mRNA.	2,04390663	< 0,00005
G6PD	glucose-6-phosphate dehydrogenase (G6PD), transcript variant 1, mRNA.	2,04358684	< 0,0005
HCFC1R1	host cell factor C1 regulator 1 (XPO1 dependent) (HCFC1R1), transcript variant 3, mRNA.	2,040033181	< 0,0005
IGFBP2	insulin-like growth factor binding protein 2, 36kDa (IGFBP2), mRNA.	2,037895127	< 0,00005
SERF2	small EDRK-rich factor 2 (SERF2), mRNA.	2,037802689	< 0,00005
TIMP1	TIMP metallopeptidase inhibitor 1 (TIMP1), mRNA.	2,033592444	< 0,00005
C1ORF21	chromosome 1 open reading frame 21 (C1orf21), mRNA.	2,032296492	< 0,0005
ABHD14A	abhydrolase domain containing 14A (ABHD14A), mRNA.	2,029186393	< 0,00005
ERRFI1	ERBB receptor feedback inhibitor 1 (ERRFI1), mRNA.	2,028448223	< 0,00005
HNMT	histamine N-methyltransferase (HNMT), transcript variant 1, mRNA.	2,026724432	< 0,05
C7ORF41	chromosome 7 open reading frame 41 (C7orf41), mRNA.	2,023097201	< 0,00005
PARC	p53-associated parkin-like cytoplasmic protein (PARC), mRNA.	2,020116731	< 0,05
NOL12	nucleolar protein 12 (NOL12), mRNA.	2,018538704	< 0,00005
C5ORF4	chromosome 5 open reading frame 4 (C5orf4), transcript variant 1, mRNA.	2,018122943	< 0,0005
ZSCAN18	zinc finger and SCAN domain containing 18	2,016589496	< 0,00005

	(ZSCAN18), mRNA.		
RSPO3	R-spondin 3 homolog (Xenopus laevis) (RSPO3), mRNA.	2,015026432	< 0,0005
SLC43A3	solute carrier family 43, member 3 (SLC43A3), mRNA.	2,013588961	< 0,05
ARL6IP5	ADP-ribosylation-like factor 6 interacting protein 5 (ARL6IP5), mRNA.	2,012197737	< 0,00005
CCL8	chemokine (C-C motif) ligand 8 (CCL8), mRNA.	2,010108639	< 0,0005
CCL13	chemokine (C-C motif) ligand 13 (CCL13), mRNA.	2,005080439	< 0,005
CCR2	chemokine (C-C motif) receptor 2 (CCR2), transcript variant A, mRNA.	2,004342656	< 0,005
